# The endoplasmic reticulum degradation-enhancing α-mannosidase-like protein 3 attenuates the unfolded protein response and has pro-survival and pro-viral roles in hepatoma cells and hepatocellular carcinoma patients

**DOI:** 10.1186/s12929-024-01103-9

**Published:** 2025-01-22

**Authors:** Alina-Veronica Ghionescu, Mihaela Uta, Andrei Sorop, Catalin Lazar, Petruta R. Flintoaca-Alexandru, Gabriela Chiritoiu, Livia Sima, Stefana-Maria Petrescu, Simona Olimpia Dima, Norica Branza-Nichita

**Affiliations:** 1 Department of Viral Glycoproteins, Institute of Biochemistry of the Romanian Academy, Splaiul Independentei 296, Sector 6, 060031 Bucharest, Romania; 2 Center of Excellence in Translational Medicine, Fundeni Clinical Institute, Soseaua Fundeni 258, Sector 2, 022328 Bucharest, Romania; 3 Department of Molecular Cell Biology, Institute of Biochemistry of the Romanian Academy, Bucharest, Romania; 4 Digestive Diseases and Liver Transplantation Center, Fundeni Clinical Institute, Soseaua Fundeni 258, Sector 2, 022328 Bucharest, Romania

**Keywords:** ER degradation, HBV infection, Cancer, Autophagy

## Abstract

**Background:**

Chronic hepatitis B virus (HBV) infection is a major risk for development of hepatocellular carcinoma (HCC), a frequent malignancy with a poor survival rate. HBV infection results in significant endoplasmic reticulum (ER) stress and activation of the unfolded protein response (UPR) signaling, a contributing factor to carcinogenesis. As part of the UPR, the ER**-**associated degradation (ERAD) pathway is responsible for removing the burden of misfolded secretory proteins, to re**-**establish cellular homeostasis. Emerging evidence indicates consistent upregulation of ERAD factors, including members of the ER degradation**-**enhancing alpha**-**mannosidase**-**like protein (EDEM) family in infection and various tumor types. However, the significance of this gene expression pattern in HBV-driven pathology is just beginning to be deciphered.

**Methods:**

In this study we quantified the expression of the ERAD factor EDEM3, in a cohort of HCC patients with and without HBV infection, and validated our results by analysis of publically available transcriptomic and microarray data sets. We performed mechanistic studies in HepaRG cells with modulated EDEM3 expression to address UPR, ERAD, autophagy and apoptosis signaling, and their consequences on HBV infection.

**Results:**

Our work revealed significantly elevated EDEM3 expression in HCC tissues irrespective of HBV infection, while the highest levels were observed in tissues from HBV**-**infected patients. Investigation of published transcriptomic data sets confirmed EDEM3 upregulation in independent HCC patient cohorts, associated with tumor progression, poor survival prognosis and resistance to therapy. EDEM3**-**overexpressing hepatic cells exhibited attenuated UPR and activated secretory autophagy, which promoted HBV production. Conversely, cell depletion of EDEM3 resulted in significant ER stress inducing pro**-**apoptotic mechanisms and cell death.

**Conclusions:**

We provide evidence of major implications of the ERAD pathway in HBV infection and HCC development and progression. Our results suggest that ERAD activation in HBV**-**infected cells is a protective mechanism against prolonged ER stress, potentially contributing to establishment of chronic HBV infection and promoting tumorigenesis. Developing specific inhibitors for ERAD factors may be an attractive approach to improve efficiency of current antiviral and anticancer therapies.

**Supplementary Information:**

The online version contains supplementary material available at 10.1186/s12929-024-01103-9.

## Background

Hepatitis B virus (HBV) is a life-threatening human pathogen at global level, affecting more than 296 million people and causing 1.5 million new infections each year [1]. Chronically**-**infected patients are at high risk of developing cirrhosis and hepatocellular carcinoma (HCC) which leads to about 1 million deaths annually [2]. During HBV infection, a vast quantity of the large (L), medium (M) and small (S) envelope glycoproteins is synthesized in the endoplasmic reticulum (ER) of the host cells, where they undergo complex disulfide bonding and oligomerization. A minor fraction of these proteins is used for the envelopment of mature nucleocapsids, the resulting virus particles being released via the multivesicular bodies (MVBs)/secretory autophagy pathway [3]; the remaining excess of surface proteins is assembled in non-infectious subviral particles (SVPs), collectively known as hepatitis B surface antigen (HBsAg), and secreted by the constitutive secretory pathway [4].

In vitro studies have indicated that HBV infection can induce significant ER stress and stimulate the early stages of non**-**degradative autophagy to support own replication and secretion [5]. The ER accumulation of HBsAg is the major trigger of the ER stress, resulting in unfolded protein response (UPR) signaling, impaired degradative autophagy and increased cell proliferation. These are aggravating factors of the liver pathology, eventually driving hepatocarcinogenesis, as evidenced in vivo [6]. The UPR is an adaptive mechanism activating a number of ER resident proteins, interconnected with the protein quality control and the ER-associated degradation (ERAD) pathways, to re-establish cellular homeostasis. These molecular sensors, including inositol-requiring enzyme 1α (IRE1α), activating transcription factor 6 (ATF6) and double-stranded RNA-activated protein kinase (PKR)-like ER kinase (PERK) [7, 8], act through different mechanisms to remove misfolded proteins, increase the folding capacity in the ER and reduce protein translation. However, if the UPR fails to mitigate the ER stress, when this is persistent or critical, the same pathways may promote senescence and ultimately apoptotic cell death [8].

As an integral part of the UPR, ERAD is responsible for the clearance of terminally misfolded proteins by cytosolic proteasomal degradation [9, 10]. This process starts within the ER with the sequential removal of mannose residues from the N-linked oligosaccharides attached to misfolded protein substrates, by the ER degradation-enhancing alpha-mannosidase-like family of proteins (EDEM1, 2, 3) [11, 12]. Such oligosaccharide trimming provides the appropriate molecular signals to irreversibly engage the misfolded glycoproteins in the degradation pathway, following their retro-translocation to the cytosol [13].

We have previously shown that EDEMs are significantly upregulated in HBV-replicating cells, in response to the ER stress induced by the accumulation of the viral envelope glycoproteins [14]. The N-linked glycans attached to the envelope proteins are processed by EDEMs (more efficiently by EDEM3), resulting in degradation of L and S, when expressed independently; in striking contrast, the M protein not only is spared from disposal but its trafficking is accelerated, leading to improved secretion [15]. These intriguing effects prompted us to investigate further the role of ERAD in the HBV life cycle, in HCC patients and cellular systems permissive for productive infection, previously unexplored. For detailed analysis we have particularly focused on EDEM3, as a representative of the EDEM family, with overlapping, but more efficient α-1,2 mannosidase activity than EDEM1 [12]. Our investigation revealed elevated EDEM3 levels in HCC tissues regardless of the underlying etiology and a significantly increased expression in tumors derived from HBV-infected patients. Analysis of publically available transcriptomic data sets confirmed the high EDEM3 expression in independent HCC patient cohorts, associated with tumor progression, poor survival prognosis and resistance to therapy. Mechanistic studies performed in HepaRG cells showed that EDEM3 overexpression results in attenuation of the UPR and stimulation of non-degradative autophagy, which promotes HBV production. In turn, significant UPR activation and apoptosis occur in EDEM3 knockout cells. Together, our data suggest that ERAD activation induced in HBV**-**infected cells may function as a cyto**-**protective mechanism against long**-**term ER stress, contributing to HBV chronicity and ultimately tumorigenesis.

## Methods

### Cell culture, generation of cell lines and nucleic acid transfections

HepaRG cells (kind gift from Dr. David Durantel, INSERM U871, Lyon, France) were grown in William’s E Medium GlutaMAX supplemented with 10% fetal bovine serum (FBS), 50 U/mL penicillin–streptomycin (all from Gibco, Paisley, UK), 5 μg/mL insulin and 5 × 10^–5^ M hydrocortisone hemisuccinate (both from Sigma-Aldrich, St. Louis, MO, USA), as described [16]. The HepaRG cell line overexpressing EDEM3 was generated using an amphotropic retroviral system. Briefly, the retroviral vector pLPCX (Clontech, Palo Alto, CA, USA), containing the EDEM3 DNA sequence, and the corresponding control vector lacking this sequence [17], were co-transfected with pCL-Ampho (Imgenex, San Diego, CA, USA) packaging vector into HEK293T cells, and the resulting retroviral particles were used to transduce HepaRG cells. To knock-out EDEM3 expression, HepaRG cells were co-transfected with clustered regularly interspaced palindromic repeats (CRISPR)/CRISPR associated protein 9 (Cas9) (sc-408476, Santa Cruz Biotechnology, Dallas, TX, USA) and homology-directed repair (HDR) (sc-408476-HDR, Santa Cruz Biotechnology) plasmids. Cell colonies selected in the presence of 1 µg/mL puromycin (Invivogen, Toulouse, France) were further expanded and denoted HepaRG^C^, HepaRG^EDEM3^ and HepaRG^EDEM3KO^, to indicate no changes in EDEM3 expression (control), overexpression, and knock-out, respectively. Additional control HepaRG cell lines overexpressing EDEM1 and EDEM2 were obtained using the same retroviral system [18], further denoted as HepaRG^EDEM1^ and HepaRG^EDEM2^. HepG2 2.2.15 cells stably transfected with two copies of the HBV genome (kind gift from Dr. David Durantel), were grown as described [19]. Plasmids encoding for ERAD substrates, pcDNA3.1-BACE476 (kind gift from Dr. Maurizio Molinari, Institute for Research in Biomedicine, Bellinzona, Switzerland) and pTriExTyrST, expressing a truncated Beta**-**site APP-cleaving enzyme 1 (BACE) isoform [20] and a soluble tyrosinase [21], respectively, were used to investigate protein degradation. Plasmid pTriExHBV1.1 containing 1.1 units of the HBV genome was described before [22]. pEGFPC1-LC3 plasmid [14] was used to investigate the autophagy process. All transfections were performed by using Lipofectamine 3000 (Invitrogen, Carlsbad, CA, USA). Where indicated, cells were treated with 2.5 µg/mL tunicamycin (TM, sc-3506A, Santa Cruz Biotechnology), for 6 h, to induce the ER stress.

### Cycloheximide (CHX) chase assay

To monitor degradation kinetics of a typical ERAD substrate, HepaRG^C^ and HepaRG^EDEM3^ cells were transfected with pcDNA3.1-BACE476 for 48 h. The cell culture medium was supplemented with 50 μM CHX (239763-M, Sigma-Aldrich) for the indicated time points.

### Clinical specimens

The study included a total of 60 patients (retrospective cohort): 50 with HCC (of which 25 associated with chronic HBV infection and 25 negative for viral infections) and 10 with liver-related benign pathologies, who underwent curative liver treatment at Fundeni Clinical Institute (Bucharest, Romania). Samples consisting of 50 tumor tissue (T), 50 normal adjacent to tumor tissue (NAT) and 10 normal (N) liver tissues were collected at the time of surgery in RNA stabilization solution (Sigma-Aldrich) and stored at −80 °C for further analysis. Their clinical features are listed in Supplementary Tables 1, 2.

### Antibodies

Primary antibodies used for western blot analyses include mouse anti-EDEM3 (E0409, Sigma-Aldrich, 1:500), rabbit anti-EDEM1 (E8406, Sigma-Aldrich, 1:1000), rabbit anti-EDEM2 (JD-32) (sc**-**130460, Santa Cruz Biotechnology, 1:250), rabbit anti-HBsAg (NB100-62652, Novus Biologicals, Littleton, CO, USA, 1:1000), rabbit anti-phospho-IRE1 alpha (Ser724) (PA1**-**16927, Invitrogen, 1:1000), rabbit anti-IRE1α (14C10, Cell Signaling Technology, Danvers, MA, USA, 1:1000), mouse anti**-**ATF6 (70B1413.1, Novus Biologicals, 1:1000), anti-glucose-regulated protein 94 (GRP94; H-10, Santa Cruz Biotechnology, 1:500), rabbit anti**-**phospho**-**PERK-T980 (16F8, Cell Signaling Technology, 1:1000), rabbit anti-PERK (C33E10, Cell Signaling Technology, 1:1000), rabbit anti**-**phospho-alpha subunit of eukaryotic initiation factor 2, S51(p**-**eIF2α; 9721S, Cell Signaling Technology, 1:1000), rabbit anti-eIF2α (9722S, Cell Signaling Technology, 1:1000), mouse anti-binding immunoglobulin protein (GRP78/BiP; A-10; sc-376768, Santa Cruz Biotechnology, 1:200), rabbit anti-suppressor of Lin**-**12**-**like (Sel1L; ab78298, Cell Signaling Technology, 1:500), rabbit anti-HMG-CoA reductase degradation protein 1, also known as Synoviolin (Hrd1; 14773, Cell Signaling Technology, 1:1000), rabbit anti-ER lectin 1 (XTP3-B/ERLEC1; ab181166, Abcam, Cambridge, UK, 1:500), rabbit anti-osteosarcoma amplified**-**9 (OS-9; ab19853, Abcam, 1:500), rabbit anti-Ras homolog enriched in the brain (Rheb; E1G1R, Cell Signaling Technology, 1:1000), goat anti-apolipoprotein E (ApoE; AB947, Sigma-Aldrich, 1:5000), rabbit anti-microtubule-associated protein light chain 3A/B (LC3A/B; D3U4C, Cell Signaling Technology, 1:1000), rabbit anti-phospho-mammalian target of rapamycin (mTOR Ser2448; D9C2, Cell Signaling Technology, 1:1000), rabbit anti-mTOR (7C10, Cell Signaling Technology, 1:1000), mouse anti-p53 (DO-1, sc-126, Santa Cruz Biotechnology, 1:500), mouse anti-pro-apoptotic effector B-cell lymphoma protein 2 (Bcl-2) Associated X (BAX; MA5-14003, Invitrogen, 1:500), rabbit anti-calnexin (ab22595, Abcam, 1:5000), mouse anti**-**β**-**actin (ab8224, Abcam, 1:5000), rabbit anti-glyceraldehyde 3-phosphate dehydrogenase (GAPDH; PA1-987, Invitrogen, 1:5000), rabbit anti-α-tubulin (ab6046, Abcam, 1:10000). Secondary antibodies used for western blot analyses include goat anti-rabbit (sc-2004, Santa Cruz Biotechnology, 1:10000), mouse IgGκ light chain binding protein (sc-516102, Santa Cruz Biotechnology, 1:10000), donkey anti-goat (sc-2020, Santa Cruz Biotechnology, 1:10000). For immunofluorescence microscopy, we used the following antibodies: rabbit anti-EDEM3 (E8906, Sigma-Aldrich, 1:300), rabbit anti-sodium taurocholate co-transporting polypeptide (NTCP; sc-98484, Santa Cruz Biotechnology, 1:100), mouse anti-protein disulfide isomerase (PDI; ab52587, Abcam, 1:1000) or mouse anti-albumin (sc-2716051, Santa Cruz Biotechnology, 1:50), followed by incubation with Alexa Fluor 488-conjugated, goat anti-rabbit (A-11008, Invitrogen, 1:400) or Alexa Fluor 594-conjugated, donkey anti-mouse (A-21203, Invitrogen, 1:400) secondary antibodies. In immunoprecipitation experiments mouse anti-preS1 (sc-57 762, Santa Cruz Biotechnology, 1:1000) were used.

### RNA purification and quantification by reverse-transcription (RT) real-time polymerase chain reaction (PCR)

Total RNA was isolated from liver tissue samples by using TRIzol (Invitrogen), according to the manufacturer’s instructions. RNA purity and concentration were measured with a NanoDrop ND-1000 Spectrophotometer (Thermo Fisher Scientific, Wilmington, DE, USA). cDNAs were synthesized from purified RNA by using the High-Capacity cDNA Reverse Transcription Kit (Applied Biosystems, Foster City, CA, USA) then amplified by using SYBR Green PCR Master Mix (Applied Biosystems) and specific primers, as indicated in Supplementary Table 3*.* TATA-Box Binding Protein (TBP) and GAPDH were used as reference genes for data normalization.

For in vitro experiments, the cDNAs were obtained by using the SuperScript III First-Strand Synthesis SuperMix (Invitrogen) then subjected to real-time PCR, as above. GAPDH and β-actin were also amplified as control for data normalization [16]. DNA amplification was carried out using the 7300 Real**-**Time PCR System (Applied Biosystems) or the Corbett Rotor-Gene 6000 real**-**time PCR instrument (Qiagen, Hilden, Germany). The relative gene expression was calculated using the 2^−ΔCt^ method [23, 24].

### Western blotting

Protein extracts from cells and tissue samples were prepared as described before [25]. Equal amounts of proteins were subjected to sodium dodecyl sulfate polyacrylamide gel electrophoresis then transferred to polyvinylidene difluoride or nitrocellulose membranes, by using a semi**-**dry blotter (Bio**-**Rad, Hercules, CA, USA). Membranes were further incubated with blocking buffers and proteins of interest were detected with corresponding antibodies according to the manufacturer’s instructions. Protein signals were visualized by using an enhanced chemiluminescence kit (ECL) or SuperSignal West Femto (Thermo Fisher Scientific**-**Pierce, Waltham, MA, USA) and quantified with the ImageJ Software (National Institutes of Health, Bethesda, MD, USA).

### HBV infection of HepaRG cells

Supernatants collected from HBV**-**replicating HepG2.2.2.15 cells were subjected to ultracentrifugation over a 20% sucrose (Sigma-Aldrich) cushion. The 300-fold-concentrated pellet containing viral particles was resuspended in phosphate buffered saline (PBS, Gibco), quantified by real-time PCR as described [14] and used to infect differentiated HepaRG cells, following published protocols. For HBV internalization experiments, infected cells were collected after 24 h, as described before [16]. When indicated, 20 µM kifunensine (Kif, sc-201364A, Santa Cruz Biotechnology) was added to the cells at different days post-infection (dpi). Cells and supernatants were collected at different dpi, and analyzed as indicated.

### Enzyme-linked immunosorbent assay (ELISA)

Secretion of SVPs from HBV-infected HepaRG cells was quantified using the Monolisa HBsAg Ultra Kit (Bio-Rad), following the manufacturer’s instructions. Plates were read using the Mithras LB940 Microplate Reader (Berthold Technologies, Bad Wildbad, Germany). Results were represented as percentage of HBsAg secretion relative to control samples.

### In-cell ELISA

Differentiated HepaRG cells were grown in 96-well plates and fixed with 4% paraformaldehyde (PFA, Sigma-Aldrich) for 5 min at room temperature. Cells were then washed with HBSS (Hank’s Balanced Salt Solution, Corning, NY, USA) followed by 50 mM glycine (Sigma-Aldrich), then permeabilized with 0.2% Triton X-100 in HBSS for 5 min. Cells were then blocked with 5% skimmed milk (Sigma-Aldrich) in HBSS for 1 h at room temperature and incubated overnight at 4 °C with rabbit anti-NTCP antibody (PA5-25614, Invitrogen, dilution 1:50). The plate was incubated with horseradish peroxidase (HRP)-labeled donkey anti-rabbit secondary antibody (31458, Invitrogen, dilution 1:1000) for 1 h at room temperature followed by addition of the HRP substrate, 3,3′,5,5′ tetramethylbenzidine (BD Biosciences, San Jose, CA, USA). The reaction was stopped with sulfuric acid and the plate was read using the Mithras LB940 Microplate Reader.

### Immunoprecipitation of enveloped HBV and DNA quantification by real-time PCR

Cell supernatants (500 µL) were incubated with anti-preS1 antibodies and 10 µL of protein G- Sepharose beads (Invitrogen), overnight, at 4 °C, to precipitate enveloped HBV particles. After washing five times with PBS, the bound virions were eluted with 50 mM Tris–HCl buffer, pH 8, containing 1 mM ethylenediaminetetraacetic acid (EDTA, Sigma-Aldrich) and 1% Nonidet P-40 (Sigma-Aldrich). The encapsidated viral DNA was quantified as described [14].

### Immunofluorescence microscopy

Cells were seeded on collagen-coated, 19 mm-diameter coverslips and fixed with 4% PFA (Sigma-Aldrich) for 20 min at room temperature. Next, the cells were permeabilized with 0.1% saponin (Sigma-Aldrich) and 3% bovine serum albumin (BSA, Sigma-Aldrich) in PBS, for 60 min at room temperature, followed by incubation with appropriate antibodies. For LC3 visualization, cells were first transfected with pEGFPC1-LC3 [14], and treated or not with 10 µM chloroquine (CQ, Sigma-Aldrich), for 24 h. All antibodies were diluted in PBS containing 0.1% saponin or 3% BSA, according to the manufacturer’s instructions. Cell nuclei were stained with 4′6-diamidino-2-phenylindole (DAPI, Thermo Fisher Scientific). Samples were visualized with either Zeiss Axio Imager M2 Fluorescence Microscope, Zeiss AxioImager.Z1 inverted microscope, or Zeiss LSM 710 microscope (63×, oil, 1.4 numerical aperture). Zen (Carl Zeiss Microscopy Deutschland GmbH, Oberkochen, Germany) and ImageJ software were further used to acquire and process the images, respectively.

### Analysis of cell apoptosis by flow cytometry

Cells were seeded on 12-well plates (10^4^ per well) and left overnight to attach. After 24 h, the supernatants were collected, cells were briefly washed with PBS (Gibco) and detached by treatment with trypsin-0.05% EDTA (Gibco). The resulting cell suspension was mixed with supernatants and washing fractions from previous steps and cells were collected by centrifugation. Apoptotic and necrotic cells were labeled using Annexin V Apoptosis Detection Kit (BD Biosciences), according to the manufacturer’s instructions. Data were acquired using the FACSVerse flow cytometer (BD Biosciences) and analyzed with the Cytobank platform (https://community.cytobank.org/).

### Transcriptomic data acquisition and processing

The transcriptomic datasets were obtained from the Gene Expression Omnibus database (GEO, https://www.ncbi.nlm.nih.gov/gds) using the GEOquery package [26] and The Cancer Genome Atlas (TCGA, https://portal.gdc.cancer.gov/) using TCGAbiolinks package [27] in the R program (version 4.3.1, https://www.R-project.org/, Vienna, Austria). The prognostic information on TCGA-Liver Hepatocellular Carcinoma (LIHC) samples (https://portal.gdc.cancer.gov/projects/TCGA-LIHC) was acquired from the University of California, Santa Cruz (UCSC) Xena database (https://xenabrowser.net/) [28]. The University of Alabama at Birmingham Cancer data analysis Portal (UALCAN, https://ualcan.path.uab.edu/) was used to process the data published by the TCGA-LIHC (NAT = 50, T = 371) and the Clinical Proteomic Tumor Analysis Consortium (CPTAC, https://cptac-data-portal.georgetown.edu/cptacPublic) HCC cohorts (NAT = 165, T = 165), with regard to EDEM3 gene and protein expression [29, 30].

To validate EDEM3 mRNA expression levels in HCC patients, we examined two microarray datasets: GSE36376 (T = 240, NAT = 193) and GSE22058 (T = 100, NAT = 97).

We also explored mRNA changes in 6 primary human hepatocyte (PHH) models that support long-term HBV infection (28 dpi): 3 controls (Mock) and 3 HBV-infected PHH samples from the GSE183156 dataset. Hierarchical clustering was performed with the pheatmap (version 1.0.12) R package using “euclidean” clustering to evaluate row distances and “complete” the agglomeration method (the distance between the remotest elements in each cluster).

Furthermore, we evaluated EDEM3 mRNA expression in HCC patients and HCC cancer cell lines treated with sorafenib, from the GEO database (accessions GSE225537, GSE192771, and GSE109211), by using the GEOquery R package. The GSE225537 data comprise HepG2 control and sorafenib-treated cells, each analyzed in 4 biological replicates. The GSE192771 data refer to Huh7 control and sorafenib-treated cells, in 3 biological replicates. The GSE109211 was used to compare EDEM3 mRNA expression in 67 formalin-fixed paraffin-embedded tumor samples from patients responding or not to sorafenib treatment [31].

For analysis of the RNA-sequencing transcriptomic data, we used transcripts per million values to normalize the mRNA expression.

### Statistical analysis

Graphs were generated using the GraphPad Prism for Windows (v.10) software (GraphPad Software, San Diego, CA, USA). Figures show means ± standard deviations (SD). Intergroup comparisons were performed using the Student’s unpaired t-test. Statistical significance was considered for *p*-values < 0.05 in all studies. The protein bands on western blots were quantified by using the ImageJ software and normalized to corresponding controls. Other statistical analyses performed according to particular experimental conditions are indicated in figure legends.

## Results

### EDEM3 is significantly upregulated in HBV-infected cells and HCC tissues

**Fig. 1:**
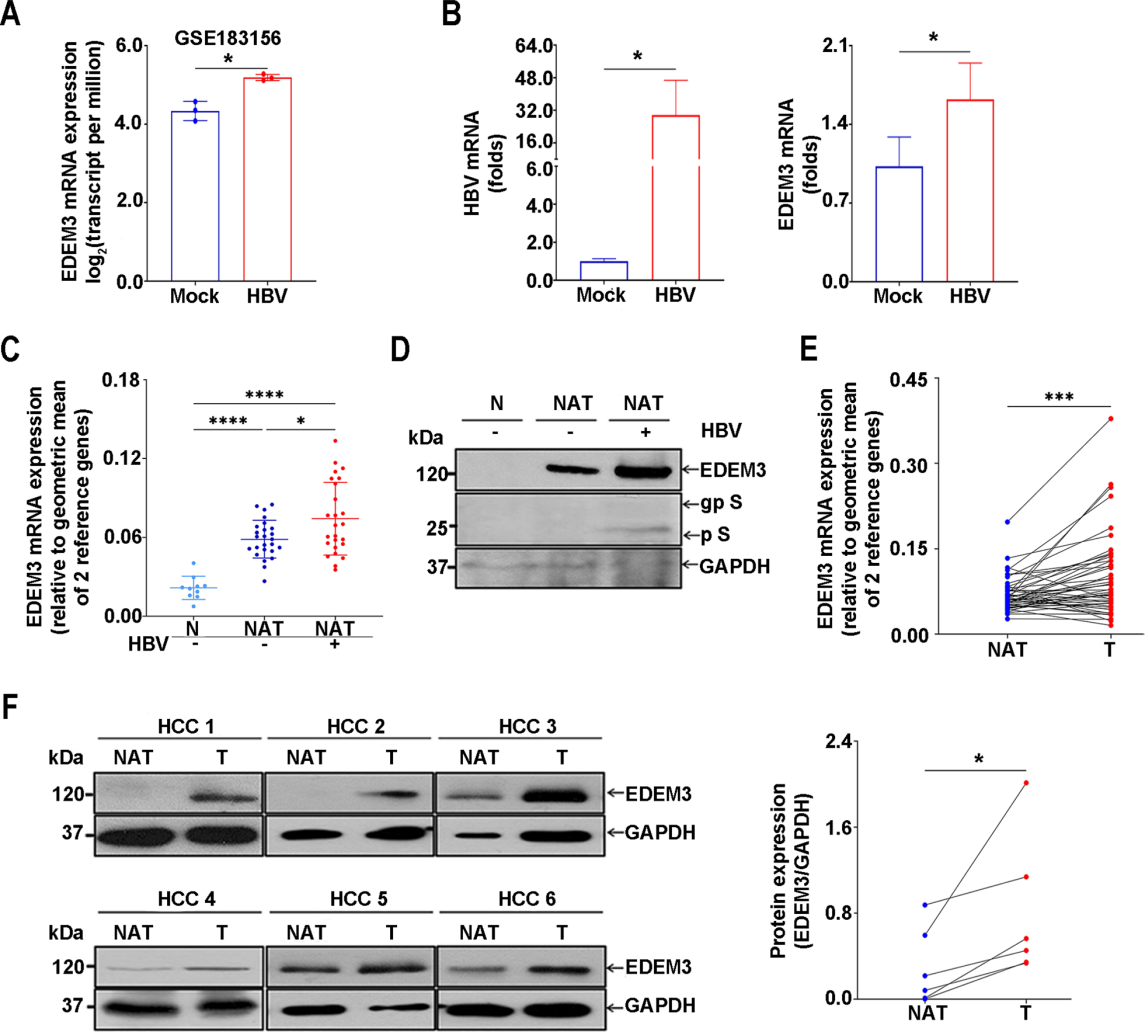
EDEM3 expression is upregulated in HBV-infected cells and HCC patient liver tissues. **A** EDEM3 mRNA expression of 3 pairs of HBV-infected PHH (HBV) and corresponding controls (Mock) at 28 dpi, based on the GSE183156 data. Statistical analysis was performed by using the unpaired t-test (*p < 0.05). **B** Total mRNA was purified from HBV-infected and control (Mock) HepaRG cells, collected at 14 dpi. HBV (left panel) and EDEM3 transcripts (right panel) were quantified by RT real-time PCR and normalized to GAPDH expression. Representative data of 2 independent experiments are shown. Statistical analysis was performed by using the unpaired t-test (*p < 0.05). **C** EDEM3 mRNA levels were quantified in 10 N and 50 NAT from two equally distributed HCC patients cohorts, without (−) or with HBV infection (+), by RT real-time PCR by using a 2^−ΔCt^ formula. Values obtained were normalized to GAPDH and TBP expression. Comparisons between groups were performed using an unpaired t-test (*p < 0.05, ****p < 0.0001). **D** EDEM3, the non-glycosylated (p S) and glycosylated (gp S) HBsAg glycoforms were detected by western blot in representative N and NAT liver tissues without (−) and with HBV (+) infection. GAPDH levels were determined as total protein loading control. **E** EDEM3 mRNA levels were determined by RT real-time PCR, normalized to GAPDH and TBP expression by using a 2^−ΔCt^ formula and compared in 50 pairs of T and NAT liver tissues. Statistical analysis was performed by using the paired t-test (***p < 0.001). **F** EDEM3 expression was examined by western blot in six pairs of T and NAT liver tissues. GAPDH levels were used as total protein loading control (left panel). The protein bands were quantified with the ImageJ Software and represented as EDEM3/GAPDH ratios (right panel). The data were analyzed using the paired t-test (*p < 0.05)

To uncover the complex signaling within the ER pathways during HBV infection, we initially investigated publically available PHH transcriptomic data sets, using an UPR gene signature. The analysis of the transcription patterns in HBV-infected PHH from the GSE183156 dataset revealed 18 upregulated UPR genes, including ERAD and autophagy markers, when compared with uninfected cells (Supplementary Fig. 1A). Interestingly, among the UPR factors, transcription of the EDEM family of genes was increased mostly in infected PHH, with EDEM3 elevated consistently and significantly, in all biological replicates (Fig. 1A). This is in agreement with previously published data, indicating increased EDEM levels in tumor cell lines supporting high HBV replication rates [14] and in HBV-infected, as compared with uninfected HCC liver tissues [32].

We therefore focused our next studies on EDEM3, as a representative member of the family, to investigate in more detail the significance of this expression profile in HBV infection. In our experiments, we used the hepatic progenitor cells HepaRG that express hepatocyte-specific markers similar to PHH and become permissive to HBV infection following differentiation [33]. Although efficiency of infection is lower in these cells than in other HBV infectivity models that are based on overexpression of the NTCPreceptor [34], the use of the HepaRG cells is desirable for investigation of complex host–pathogen interactions, due to their expression of key molecules of the innate immune system that are relevant to infection.

Quantification of viral and EDEM3 transcripts in differentiated HepaRG cells revealed significantly increased EDEM3 expression in HBV-infected cells at 14 dpi, when compared with mock-infected controls (Fig. 1B). To determine whether the HBV infection-EDEM3 relationship can be validated in more relevant clinical samples, we quantified the EDEM3 mRNA in tissues from our cohort of patients, including 10 normal, 25 HBV-infected (+) and 25 uninfected (−) normal adjacent to tumor HCC livers (Fig. 1C). The results were further confirmed at the protein level by western blot analysis (Fig. 1D). Interestingly, while the data indicated the highest EDEM3 expression in HBV-infected tissues (Fig. 1C, D), significantly increased EDEM3 levels were also found in normal adjacent to tumor when compared to normal tissues, irrespective of viral infection (Fig. 1C).

Chronic HBV infection is a well-established liver cancer risk factor [35]. The upregulated EDEM3 expression in normal adjacent to tumor compared to normal tissues suggests that the ERAD factor may also contribute to HCC development. To investigate this hypothesis, we evaluated EDEM3 expression in 50 tumor and 50 paired-normal adjacent to tumor liver tissues from our cohort of HCC patients. As shown in Fig. 1E, the amount of EDEM3 transcripts was significantly increased in tumor samples; the results were further confirmed at protein levels in 6 paired tissues by western blot analysis (Fig. 1F). Moreover, our analysis of publically available transcriptomic and microarray data indicated similarly elevated EDEM3 levels in HCC (Supplementary Fig. 1B, C).

We further took advantage of the availability of isolated mRNA from patient samples to investigate autophagy-related genes as this process is relevant to HBV replication [36], and also other ER stress sensors. Notably, unlike in short-term HBV-infected PHH (Supplementary Fig. 1A), the expression of ATF6, an important UPR regulator, remained unchanged in patient tissues regardless of the HBV infection. Conversely, the expression of two autophagy markers, LC3 and autophagy-related gene 3 (ATG3), was significantly upregulated in normal adjacent to tumor compared with normal tissues, while HBV infection had only a limited, statistically non-significant contribution to this behavior (Supplementary Fig. 2). Taken together, these results showed the dynamic of the UPR pattern in chronic as opposed to acute HBV infection and suggest a potential involvement of EDEM3 and autophagy in supporting chronic HBV infection and tumorigenesis.

### EDEM3 overexpression attenuates the ER stress and the UPR signaling pathways in HepaRG cells

**Fig. 2:**
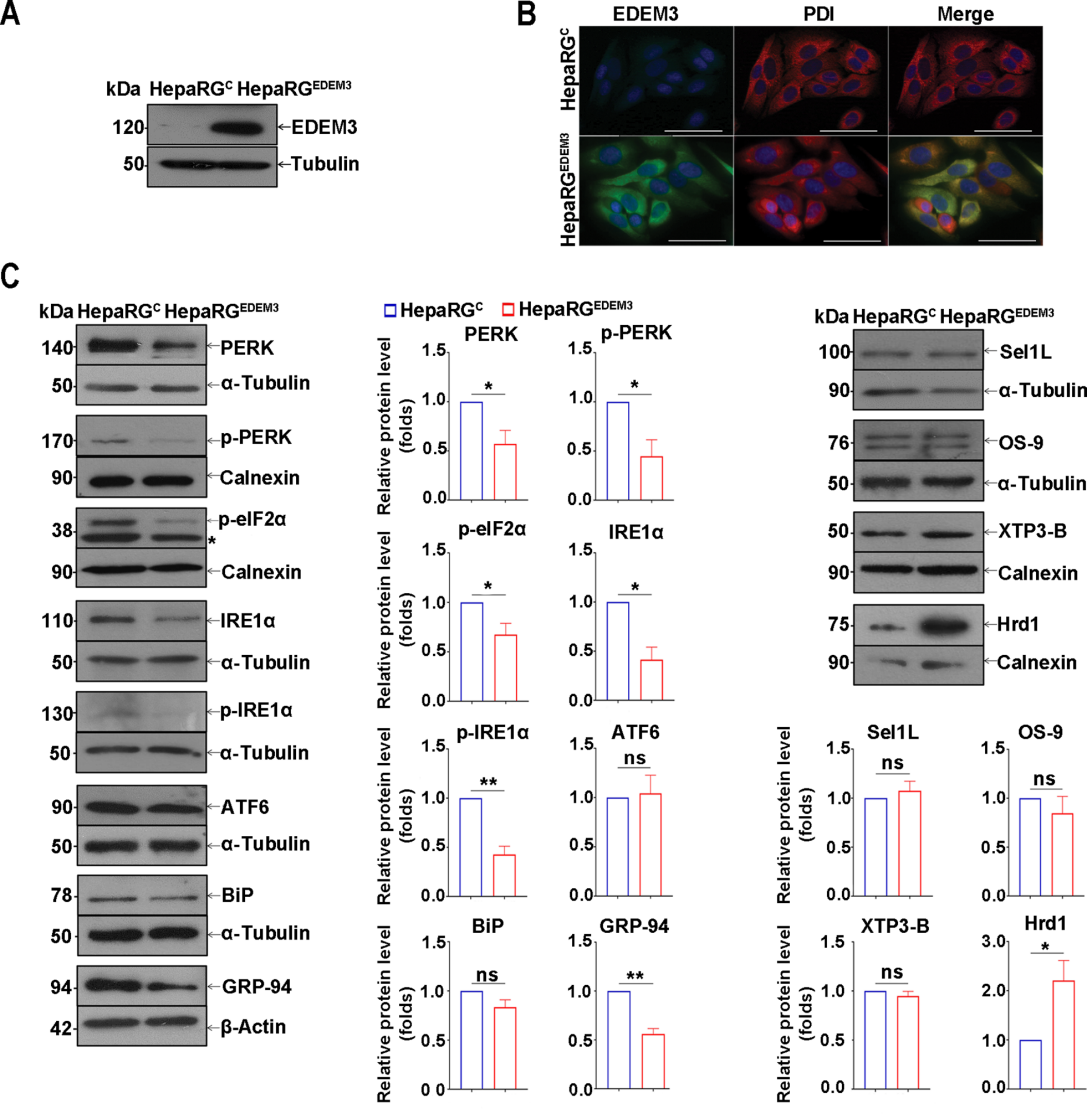
UPR pathways are downregulated in EDEM3-overexpressing HepaRG cells. **A** EDEM3 expression in HepaRG^C^ and HepaRG^EDEM3^ cells was determined by western blot. Detection of α-tubulin was used as total protein loading control. **B** Expression of EDEM3 (*green*) and PDI (*red*) was determined in HepaRG^C^ and HepaRG^EDEM3^ cells by incubation with corresponding antibodies followed by immunofluorescence microscopy. Images were analyzed with the AxioVision SE64 Rel. 4.9.1 Software. The cell nuclei were stained with DAPI (*blue*). The indicated scale bar is 50 µm. **C** Expression of UPR and ERAD markers in HepaRG^C^ and HepaRG^EDEM3^ cells was investigated by western blot with corresponding antibodies. Detection of α-tubulin, β-actin or calnexin was used as internal controls for total protein loading. The *asterisk* indicates a non-specific reactivity of the p**-**eIF2α antibody. The bands corresponding to the proteins of interest were quantified from three independent experiments by using the ImageJ software and normalized to corresponding internal controls. Statistical analysis of the relative protein expression using the unpaired t-test is shown (*p < 0.05, **p < 0.01)

Our next studies were designed to explore the mechanism of a potential association of EDEM3 levels with the development of liver disease and HBV infection, by modulating expression of the ERAD protein in HepaRG cells. As differentiated HepaRG cells are refractory to transient genetic manipulation of target genes, cell lines with stable overexpression of EDEM1-3 were produced, a strategy that has successfully been used to investigate cell factors involved in HBV infection [16, 22]. Following transduction with the genes of interest, cell colonies were selected in the presence of puromycin, expanded and characterized. Western blot analysis of selected HepaRG cells indicated high amounts of EDEM3 in the cell line transduced with the EDEM3 cDNA (HepaRG^EDEM3^), compared with control (HepaRG^C^) (Fig. 2A). This result was confirmed by fluorescence microscopy, showing the expected EDEM3 localization within the ER compartment, as revealed by significant overlapping with the ER marker, PDI (Fig. 2B).

Owing to their mannosidase activity, EDEMs provide misfolded proteins with the appropriate signal required to initiate their disposal by ERAD. This property was investigated in HepaRG ^EDEM3^ cells by monitoring the fate of well-known ERAD substrates, such as the soluble form of tyrosinase (TyrST) and truncated versions of BACE (BACE476) [20, 21]. The results indicated substantial degradation of these proteins, confirming the expression of a functional EDEM3 and an accelerated ERAD in HepaRG^EDEM3^ cells (Supplementary Fig. 3A). The results were further validated by using BACE476 as a model ERAD substrate and the CHX treatment to stop protein translation and monitor the degradation kinetics of the proteins more accurately (Supplementary Fig. 3B).

EDEM1-3 act sequentially on their substrates, the mannose trimming being initiated by EDEM2 and continued by EDEM1 and EDEM3, likely with redundant activity [37]; it was therefore important to investigate a potential modulation of endogenous EDEM1-3 synthesis in cell lines overexpressing either member of the family. The results indicated no mutual regulation of EDEM1-3 expression in HepaRG cells, suggesting that the functional effects observed can be justifiably associated with the upregulation of the corresponding EDEM protein (Supplementary Fig. 3C).

Accumulating data indicate significant activation of the ER stress, both in HBV infection and HCC development [5]. To determine the consequences of elevated EDEM3 expression on the UPR, the IRE1α, ATF6 and PERK pathways were investigated in HepaRG cells. Interestingly, while the ATF6 expression remained unchanged, the IRE1α and PERK pathways were markedly attenuated in HepaRG^EDEM3^ cells when compared with controls, as indicated by reduced phosphorylation of these stressor proteins and of their known effectors, such as the eIF2α [38] (Fig. 2C). Similarly, decreased levels of ER molecular chaperones that promote protein folding and prevent aggregation of misfolded polypeptides such as GRP94 and BiP [38] were observed in these cells (Fig. 2C).

As part of the UPR, the ERAD is a tightly coordinated, multistep process, involving substrate recognition and ubiquitination for cytosolic proteasomal degradation. Following trimming by EDEMs, other lectins, i.e. OS**-**9 and XTP3**-**B, take over the unfolded glycoproteins in the ER lumen and pass them to membrane-embedded ERAD adaptors (e.g., Sel1L) [39, 40]. Sel1L binds to Hrd1, an E3 ubiquitin ligase and key component of the retro-translocation complex, enabling protein translocation from the ER [41]. Investigation of these ERAD markers in HepaRG^EDEM3^ cells revealed similar expression patterns of OS**-**9, XTP3-B and Sel1L to control cells, while the Hrd1 levels were remarkably increased (Fig. 2C). The significance of this behaviour will be discussed later in the manuscript.

Collectively, these results suggest that increased EDEM3 levels attenuate the UPR, likely preventing a prolonged ER stress and irreversible cell damage.

### EDEM3-overexpressing HepaRG cells display mild autophagic activity

**Fig. 3:**
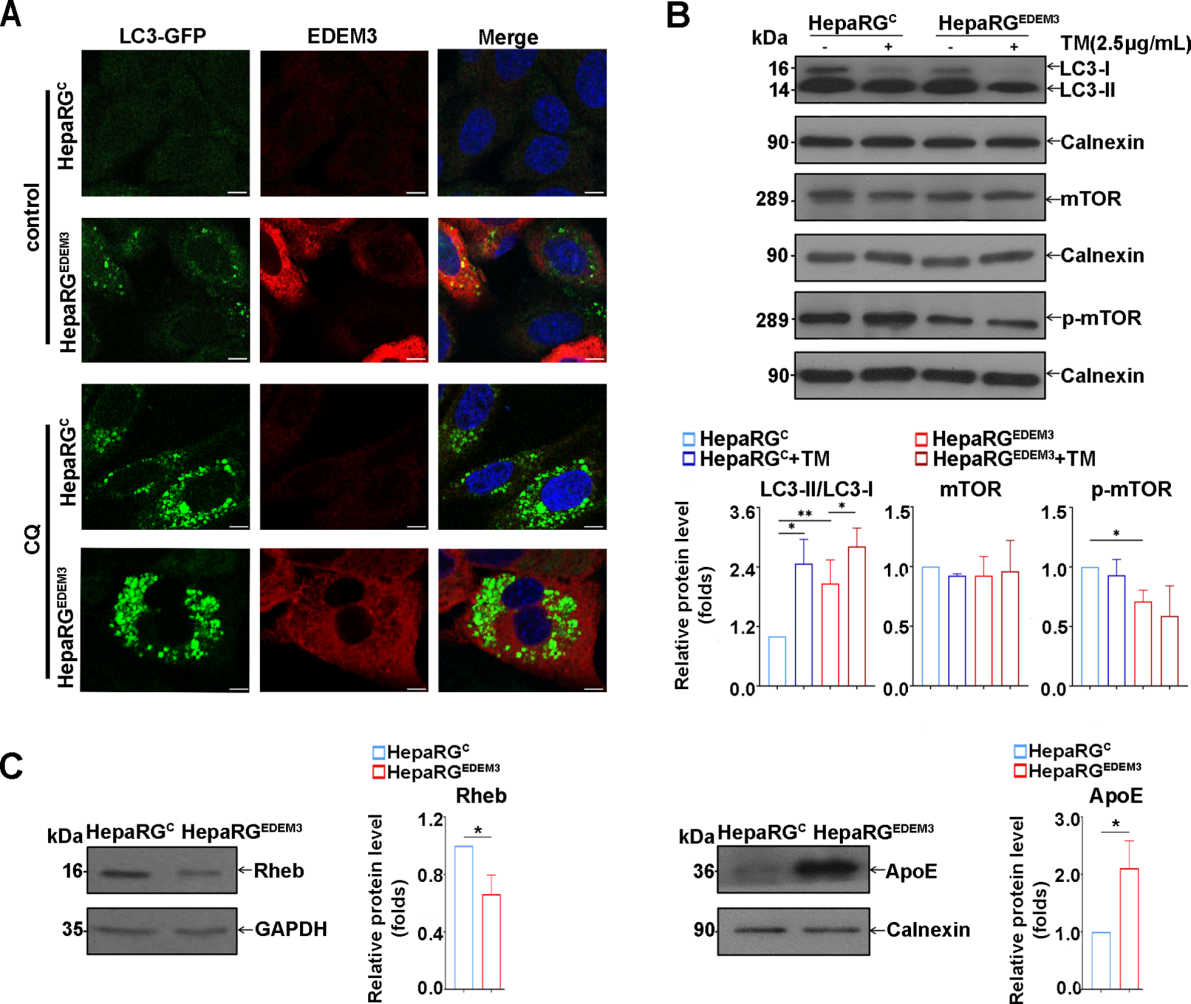
Non-degradative autophagy is increased in EDEM3-overexpressing HepaRG cells. **A** Intracellular localization of LC3 (*green*) and EDEM3 (*red*) was analyzed in HepaRG^C^ and HepaRG^EDEM3^ cells transfected with LC3-GFP, by confocal microscopy. The nuclei were stained with DAPI (*blue*). Where indicated, cells were treated with 10 µM CQ. Scale bar is 10 µm. **B**, **C** Autophagy and mTOR activation markers were investigated in HepaRG^C^ and HepaRG^EDEM3^ cells by western blot with corresponding antibodies. Where indicated, cells were treated with 2.5 µg/mL of TM for 6 h. Detection of calnexin or GAPDH was used as internal control. The bands corresponding to the proteins of interest were quantified from three independent experiments by using the ImageJ software and normalized to corresponding internal controls. Statistical analysis of the relative protein expression using the unpaired t-test is shown (*p < 0.05, **p < 0.01)

Analysis of autophagy-related genes in our patient cohort indicated considerable activation of this pathway in NAT compared to N tissues, possibly a protective mechanism in response to the dysregulated homeostasis triggered by tumorigenesis and extended beyond tumors, to adjacent areas (Supplementary Fig. 2). To determine whether EDEM3 is involved in this process, autophagy was investigated in HepaRG^EDEM3^ cells transfected with the pEGFPC1-LC3 plasmid encoding LC3, a well-established autophagy marker [42, 43]. Cell treatment with CQ, an inhibitor of the autophagic flux [44], was also included as a control. Confocal microscopy analysis indicated a high number of LC3-labelled puncta in HepaRG^EDEM3^ cells, while these were hardly detectable in untreated control cells. As expected for an ER-resident protein, EDEM3 staining did not co-localize with that of LC3. Addition of CQ significantly enhanced the LC3 fluorescence in both cell lines confirming the specificity of the assay (Fig. 3A). Consistently, an increased conversion of soluble LC3-I to lipid-bound LC3-II, recruited during autophagosome assembly, was observed in HepaRG^EDEM3^ cells by western blot (Fig. 3B). This process was further accelerated by TM, a protein N-glycosylation inhibitor and ER stress inducer, included in the experiment as an additional control [45] (Fig. 3B). Interestingly, very recently, cell treatment with low TM concentrations was also shown to mimic the ER stress induced by HBV infection in hepatic cells [5].

As mTOR is a major regulator of autophagy [46, 47], we next investigated this pathway in EDEM3-overexpressing cells. Notably, mTOR activation by phosphorylation was evidently impaired in these cells, although the overall protein level was not affected, even in the presence of TM (Fig. 3B). Consistently, expression of the Rheb GTPase, the key upstream activator of mTOR [48, 49], was down-regulated in HepaRG^EDEM3^ cells (Fig. 3C). Interestingly, it has recently been shown that Rheb is a novel ERAD substrate; following binding to Hrd1, Rheb is recruited by the Sel1L/Hrd1 complex and ubiquitinated for degradation [50]. Thus, it is tempting to hypothesize that the reduced Rheb level in HepaRG^EDEM3^ cells, is a result of the Hrd1 upregulation in these cells, as described above.

We further investigated the consequences of EDEM3 overexpression on the fate of ApoE, a protein degraded by autophagy in hepatic cells [51]. Notably, in contrast to the case of proteasomal substrates (Supplementary Fig. 3), ApoE levels were clearly higher in HepaRG^EDEM3^ cells than in control cells (Fig. 3C), indicating reduced lysosomal activity and degradation by autophagy in EDEM3-overexpressing cells. Together, our data are in support of the notion that EDEM3 may trigger non-degradative autophagy in hepatic cells, through regulation of the Hrd1/Rheb/mTOR pathway.

### EDEM3 knockout promotes apoptosis in HepaRG cells

**Fig. 4:**
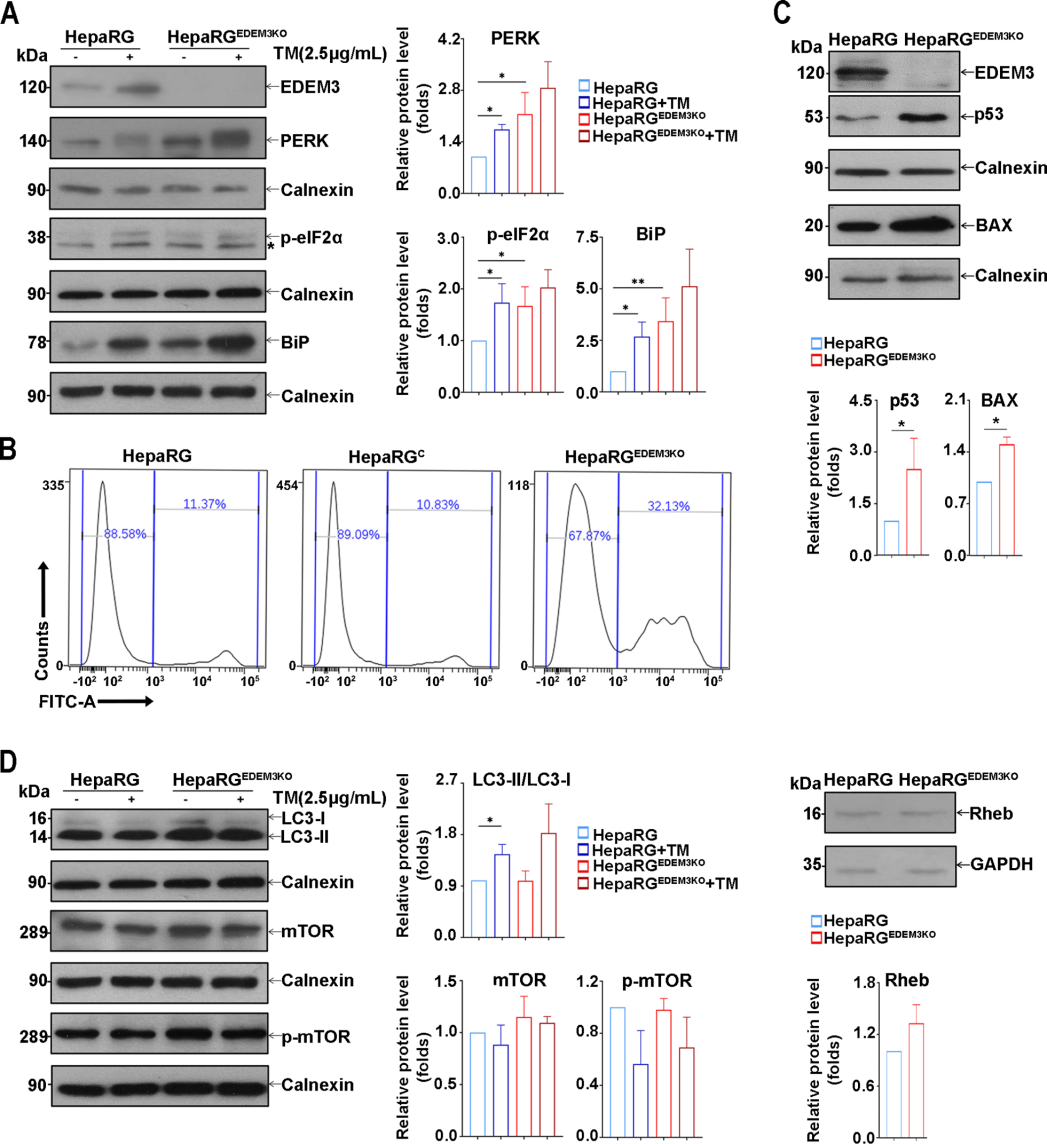
EDEM3 depletion promotes apoptosis in HepaRG cells. **A** Expression of UPR markers in HepaRG and HepaRG^EDEM3KO^ cells was determined by western blot with corresponding antibodies. Where indicated, cells were treated with 2.5 µg/mL TM for 6 h. Detection of calnexin was used as internal control. The asterisk indicates a non-specific reactivity of the p**-**eIF2α antibodies. The bands corresponding to the proteins of interest were quantified from three independent experiments by using the ImageJ software and normalized to corresponding internal controls. Statistical analysis of the relative protein expression using the unpaired t-test is shown (*p < 0.05, ***p < 0.001). **B** Cell apoptosis was evaluated by flow cytometry. Histograms indicate the percentage of viable and Annexin V+ cells, as detected on fluorescein isothiocyanate (FITC) channel. Data shown are representative of two independent experiments. **C** Expression of apoptotic markers in HepaRG and HepaRG^EDEM3KO^ cells was determined by western blot with corresponding antibodies. Detection of calnexin was used as an internal control. The bands corresponding to the proteins of interest were quantified from three independent experiments by using the ImageJ software and normalized to corresponding internal controls. Statistical analysis of the relative protein expression using the unpaired t-test is shown (*p < 0.05). **D** Autophagy and mTOR activation markers were investigated in HepaRG and HepaRG^EDEM3KO^ cells by western blot with corresponding antibodies. Where indicated, cells were treated with 2.5 µg/mL of TM for 6 h. Detection of calnexin or GAPDH was used as internal control. The bands corresponding to the proteins of interest were quantified from three independent experiments by using the ImageJ software and normalized to corresponding internal controls. Statistical analysis of the relative protein expression using the unpaired t-test is shown (*p < 0.05)

To validate the role of EDEM3 in cell fate during ER stress, we generated CRISPR/Cas9 gene-edited EDEM3 knockout HepaRG cells (HepaRG^EDEM3KO^). During amplification of this cell line, significant cell death was observed as the passage number increased (above 10). We therefore investigated the UPR signaling in these cells at lower passages (4–5), in the absence or presence of TM. Analysis of protein expression by western blot confirmed successful knockout of endogenous EDEM3 in HepaRG^EDEM3KO^ cells (Fig. 4A). Markedly up-regulated levels of BiP, the main UPR sensor, were found in the absence of EDEM3 expression, suggesting significant ER stress in these cells, while addition of TM augmented this effect, as expected. This interpretation is strongly supported by the enhanced levels of PERK and of the downstream effector, p-eIF2α in cells depleted of EDEM3 (Fig. 4A).

Since persistent ER stress and failure to restore cellular homeostasis lead to activation of pro-apoptotic mechanisms [52, 53], we further addressed the possibility that EDEM3 depletion could trigger apoptosis in hepatic cells. Apoptotic cells were detected by flow cytometry, based on recognition of phosphatidylserine exposed on the cell surface by Annexin V [54]. Notably, a higher percentage of cells engaging apoptosis were present in the HepaRG^EDEM3KO^ cell sample compared with controls (Fig. 4B, Supplementary Fig. 4). Moreover, central regulators of apoptosis signalling, such as the p53 protein and its downstream target, Bax, a member of the Bcl-2 family of cell death regulatory proteins [55], were substantially upregulated in EDEM3-depleted cells (Fig. 4C).

Analysis of the Rheb/mTOR pathway and the LC3-I to LC3-II conversion indicated no apparent changes in autophagy in HepaRG^EDEM3KO^ cells (Fig. 4D), suggesting that activation of pro-survival mechanisms is either impaired or inefficient under high levels of ER stress.

Thus, by alleviating the UPR signaling, EDEM3 expression appears essential to prevent cells from triggering apoptotic mechanisms under prolonged ER stress conditions.

### HBV infection and replication are markedly increased in EDEM3-overexpressing HepaRG cells

**Fig. 5:**
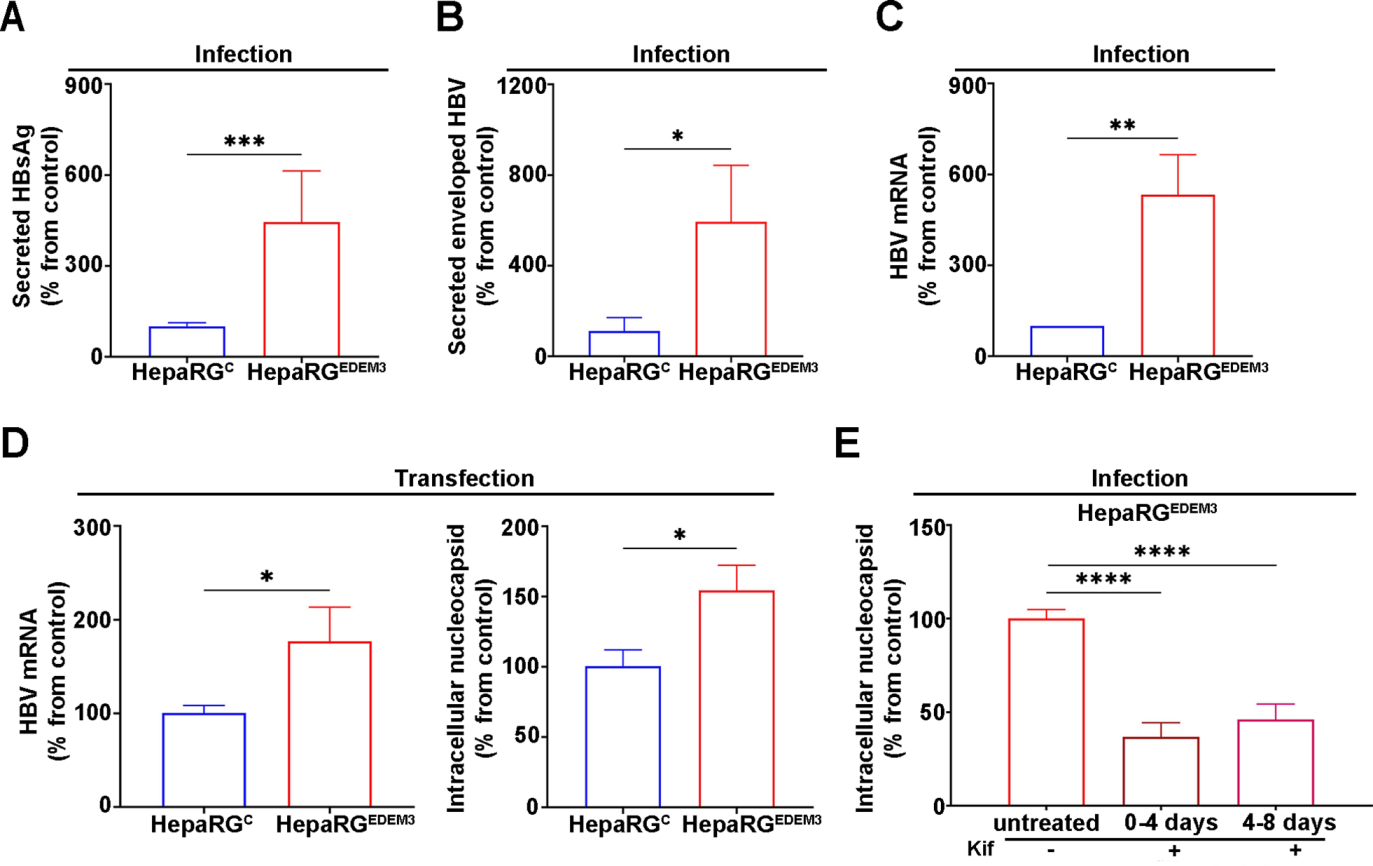
HBV infection increases in EDEM3-overexpressing HepaRG cells. Supernatants of HBV-infected HepaRG^EDEM3^ and HepaRG^C^ cells were harvested at 14 dpi and analyzed by ELISA (**A**) or subjected to immunoprecipitation with anti-preS1 antibodies, followed by viral DNA purification and quantification by real-time PCR (**B**). The results represent the data and SD from two independent experiments. Statistical analysis was performed by using the two-tailed student t-test (*p < 0.05, ***p < 0.001). **C** Total mRNA was purified from HBV-infected HepaRG^EDEM3^ and HepaRG^C^ cells collected at 14 dpi and quantified by RT real-time PCR. Obtained values were normalized to GAPDH expression. The results represent the data and SD from two independent experiments. Statistical analysis was performed by using the unpaired t-test (**p < 0.01). **D** HepaRG^EDEM3^ and HepaRG^C^ cells were transfected with pTriExHBV1.1, followed by quantification of HBV mRNA (*left*) and intracellular HBV nucleocapsids (*right panel*) by real-time PCR. The results represent the data and SD from two independent experiments. Statistical analysis was performed by using the unpaired t-test (*p < 0.05). (**E**) HBV-infected HepaRG^EDEM3^ cells were treated with 20 µM Kif for the times indicated, or maintained untreated, as control. HBV nucleocapsids were purified from cells collected at 14 dpi and DNA was quantified by real-time PCR. The results represent the data and SD from two independent experiments. Statistical analysis was measured by using the unpaired t-test (****p < 0.0001)

Non-degradative autophagy is a key process in the HBV life-cycle, promoting assembly of HBV nucleocapsids, viral replication and secretion of HBV particles [5, 56]. Moreover, accumulating evidence indicates that the phosphatidylinositol 3-kinase (PI3K)/protein kinase B (AKT)/mTOR pathway is a negative regulator of HBV replication [57]. Inhibitors of this pathway increase HBV transcription and replication, but have no effect on viral entry [5, 58]. To investigate the consequences of regulation of these pathways by EDEM3 on the HBV life-cycle, we first examined HBV infection in HepaRG^EDEM3^ cells. Cell differentiation occurred as described for the parental cell line, with the formation of biliary- and hepatocyte-like clusters (Supplementary Fig. 5A) [59]. The hepatocyte-specific differentiation markers, albumin and aldolase B transcripts were expressed at comparable levels in HepaRG^EDEM3^ cells and the corresponding control line (Supplementary Fig. 5B). Moreover, the HBV receptor, NTCP was significantly upregulated in hepatocyte-like cells, as expected (Supplementary Fig. 5C) while expressed at similar level in both cell lines, as quantified by In-cell ELISA (Supplementary Fig. 5D). No significant differences were observed with regard to efficiency of HBV infection in control and EDEM3-overexpressing HepaRG cells, as determined by analysis of viral particles internalization (Supplementary Fig. 5E). Following these characterization experiments, we concluded that the HepaRG^EDEM3^ and HepaRG^C^ cell lines are suitable models to investigate the role of EDEM3 in HBV infection. Quantification of HBsAg and HBV DNA isolated from enveloped virions revealed significantly increased levels of both viral markers in HepaRG^EDEM3^ cell supernatants at 14 dpi, compared with controls (Fig. 5A, B). Notably, the amount of total viral RNA isolated from EDEM3-overexpressing cells was up to six-fold higher than in control cells (Fig. 5C). To further clarify the stage of the HBV life-cycle impacted by EDEM3 expression, HepaRG^EDEM3^ and HepaRG^C^ cells were transfected with the HBV genome to bypass the early steps of infection and focus on viral replication. As shown in Fig. 5D, higher amounts of viral RNA and nucleocapsids were found in HepaRG^EDEM3^ cells, suggesting that mainly viral transcription and replication benefit from enhanced autophagy/low mTOR activity, as previously documented in other cell systems [3, 5].

As HBV infection appeared to benefit from EDEM3 expression, it was of interest to investigate to what extent these effects could be reversed by removing the protein from cells. Despite many optimization attempts, the HepaRG^EDEM3KO^ cell line could not be maintained in a differentiated (non-replicating) status for long enough to complete the full HBV life-cycle of about 14 days, due to severe levels of cell death. Similarly, investigation of transient EDEM3 silencing in HepaRG cells transfected with the HBV genome was inconclusive, likely due to inefficient synchronization of HBV replication and EDEM3 depletion in the same cell. We have previously shown that the effects of EDEMs on HBV envelope proteins strongly depend on their mannosidase activity [15]. We therefore considered cell treatment with Kif, an alkaloid with potent inhibition of all class I α-1,2 mannosidases [60], as an alternative approach to protein depletion. HepaRG^EDEM3^ cells were inoculated with HBV and then incubated with the inhibitor for short intervals at different dpi, followed by quantification of HBV nucleocapsids. As shown in Fig. 5E, production of HBV nucleocapsids was substantially impaired in the presence of Kif. This inhibitory effect is in agreement with previously published data indicating a significant reduction of enveloped HBV secretion in the presence of deoxymannojirimycine (DMJ) [15], another α 1,2-mannosidase inhibitor [61]. Thus, it is plausible to assume that the pro-viral role of EDEM3 in HBV infection is mediated, at least in part, by the mannosidase activity.

### EDEM3 expression is associated with overall survival rate and sorafenib resistance in HCC patients

**Fig. 6:**
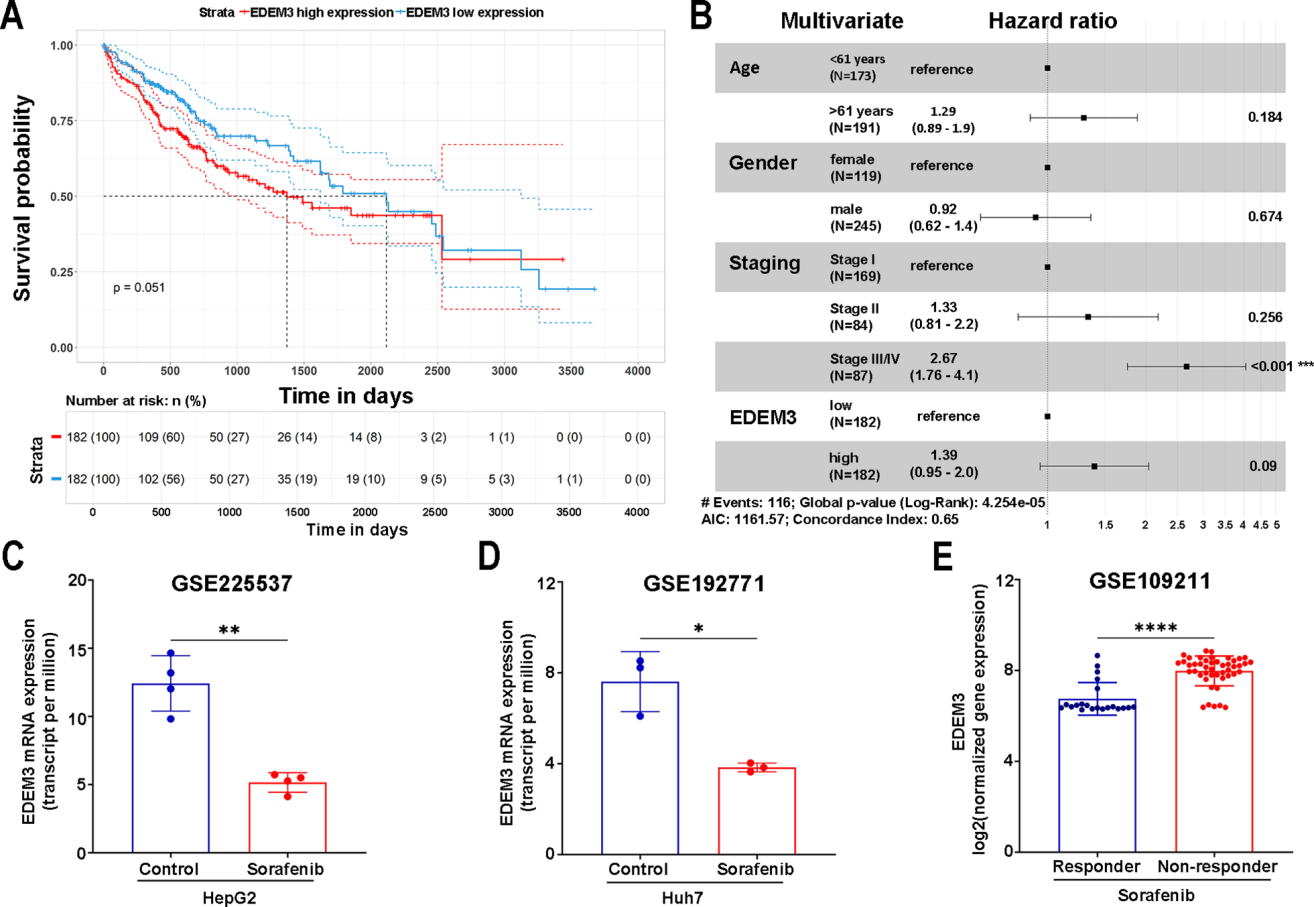
EDEM3 is correlated with treatment response in HCC patients. **A** Kaplan Meier curve shows overall survival of HCC patients from the TCGA database (n = 364), in relation to EDEM3 expression. **B** Multivariate Cox proportional hazard analysis was conducted to evaluate the hazard ratio of EDEM3 for overall survival among clinicopathological features in the TCGA-LIHC cohort. The analysis was performed by using the gtsummary 1.7.2, survival 3.5.8 and survminer 0.4.9 packages. EDEM3 expression in response to Sorafenib treatment in HepG2 cells (**C**), Huh7 cells (**D**) and HCC patients (**E**). The public databases used are shown in corresponding figures. The data were analyzed by using the unpaired t-test (*p < 0.05, **p < 0.01, ****p < 0.0001)

Our results and analysis of publically available data have clearly shown enhanced EDEM3 expression in liver tissues of HCC patients. To understand potential clinical implications of this expression pattern, we further investigated the transcriptomic data available from the TCGA-LIHC cohort. HCC patients (*n* = 364) were divided into high- and low-EDEM3 groups according to the median EDEM3 expression. The Kaplan–Meier analysis indicated that high EDEM3 expression correlated with a low survival rate (*p* = 0.051) (Fig. 6A). Nevertheless, multivariate Cox regression analysis suggested that EDEM3 is not an independent factor for survival in HCC patients (Fig. 6B). A more detailed investigation of the TCGA-LIHC cohort showed that EDEM3 was highly expressed at the initial stage of tumorigenesis and this pattern was maintained throughout more advanced stages. Besides, the subgroup analysis indicated that EDEM3 expression slightly increased according to the tumor grades, except for the undifferentiated grade IV, likely an effect of the small sample size (*n* = 12) available for this group (Supplementary Fig. 6A, B). The tumor grade takes into account the morphology of cancer cells and tissues, indicating tumor aggressiveness [62], while staging describes whether the cancer has spread to other sites in the patient’s body [63]. Thus, our analysis suggests that high EDEM3 expression may be triggered at early HCC stages, when liver damage is associated with chronic exposure to risk factors such as HBV, and is sustained regardless of the cancer progression.

The positive correlation between EDEM3 levels and patient survival rate prompted us to evaluate the mRNA expression pattern of the ERAD protein after treatment with sorafenib, a crucial chemotherapeutic agent in the clinical management of advanced HCC [64]. Sorafenib is a multiple-kinase inhibitor that suppresses tumour cell proliferation and triggers apoptosis; however, its clinical efficacy is limited due to development of drug resistance involving several mechanisms, including activation of autophagy, as recently shown [65]. Analysis of two GEO datasets (GSE225537, GSE192771), revealed a significant downregulation of EDEM3 mRNA in sorafenib-treated HepG2 (*p* < 0.01) and Huh7 cells (*p* < 0.05), when compared with untreated controls (Fig. 6C, D). This result is in support of the proposed mechanism of action of sorafenib to trigger apoptosis in hepatoma cells [66], a process that is also activated by EDEM3 depletion (our results, Fig. 4). We further performed gene expression analysis using the GSE109211 dataset [66], comprising HCC patients treated with sorafenib (*n* = 83) or placebo (*n* = 105). As shown in (Fig. 6E), EDEM3 expression is significantly higher in patients who do not respond to sorafenib treatment than in responders, suggesting a potential role in acquirement of drug resistance. Collectively, these results suggest that EDEM3 may be considered for inclusion in a panel of molecular predictors of HCC recurrence in patients receiving sorafenib.

## Discussion

Ample evidence indicates a significant disruption of the ER homeostasis in malignant cells which induces persistent ER stress and aberrant stimulation of the UPR [67]. While the initial UPR activation triggers pro-survival, adaptive mechanisms favoring tumor growth and chemoresistance, recent studies show that prolonged ER stress may steer cells towards apoptosis and death thus preventing cancer progression [68]. Therefore, it is not surprising that the UPR has been extensively investigated as a promising target in cancer therapy, a strategy that must consider the dual role of this pathway in cell survival/death in a molecular context- and tumour type-dependent manner [69, 70].

Although many studies have also highlighted the dysregulation of ERAD components in an increasing number of tumor types, the role of ERAD in cancer biology is just beginning to be disentangled. Recent evidence underlines potential roles of the ERAD factors Hrd1 and Sel1L in tumorigenesis, both independently or in association in the protein retro-translocation complex. Hrd1 overexpression in colon cancer promotes cell migration and invasion [71]. Integrative proteomic and ubiquitinomic analysis performed in tissue samples from HCC patients has indicated a significant Hrd1 upregulation and increased protein ubiquitination during cancer progression, promoting vascular invasion and metastasis [72]. Notably, well-known tumor suppressors such as phosphatase and tensin homolog (PTEN) and Wingless**-**type family member 5A (WNT5A) have been identified as novel *bona fide* Hrd1 substrates in HCC cells [73, 74]. However, their degradation has been shown to either promote [73] or suppress hepatocyte proliferation when protein disposal rescues folding deficiencies [74], indicating the molecular complexity and heterogeneity of HCC as well as the need for additional investigations.

In this study, we focused on ERAD factors acting early in the degradation pathway. We showed that EDEM3 is significantly upregulated in independent cohorts of HCC patients and that HBV infection is a contributing factor to this enhanced expression, both in vivo and in cultured cells. Hepatoma cells overexpressing EDEM3 had markedly attenuated UPR signaling, but activated autophagy, as indicated by increased LC3 lipidation and autophagosome biogenesis. In contrast, cell depletion of EDEM3 triggered significant ER stress, activation of the PERK pathway and expression of pro-apoptotic factors leading to cell death.

Mechanistically, we noted that EDEM3 expression inversely correlated with the level of Rheb and with mTOR activation. Rheb is a critical mTOR activator [48] and has recently been identified as a novel substrate of Sel1L/Hrd1-mediated degradation in hematopoietic stem cells (HSC) [50]. Rheb disposal by ERAD maintains a low mTOR activity, ensuring HSC quiescence and self-renewal [50]. In our work, markedly higher Hrd1 levels were observed in EDEM3-overexpressing HepaRG cells than in control cells, likely a positive regulatory mechanism in response to the increased degradation capacity of the ER. It is therefore conceivable that a similar inhibitory mechanism of the Rheb/mTOR axis, as depicted in HSC cells, may function in hepatoma cells with activated ERAD, resulting in stimulation of autophagy.

By using known substrates of autophagy degradation in hepatic cells [51], we were able to show that activated autophagy in the presence of EDEM3 does not necessarily result in increased protein degradation. This observation is in agreement with the data indicating pro-viral effects of EDEM3 in HBV-infected cells. Indeed, the HBV life-cycle strongly depends on different stages of autophagy [75]. Phagophores provide the membrane platform for nucleocapsid assembly and HBV replication, while autophagosomes and amphisomes are essential for core proteins trafficking and nucleocapsid delivery to MVBs for envelopment, respectively. Conversely, autolysosomes inhibit viral replication, making autophagy a central regulator of HBV infection [36]. Notably, our data indicated an enhanced production of both mature virions and SVPs in HepaRG cells overexpressing EDEM3. We have previously shown that in transiently transfected cells, a fraction of S and L envelope proteins is degraded in the presence of EDEM proteins, likely due to the incapacity of the ER to cope with the complex folding of the overexpressed proteins, while secretion of M is improved in an N-glycosylation dependent manner [15]. The lack of a detrimental effect on SVP formation in infected cells stably overexpressing EDEM3 may be a consequence of a sustained autophagy, also enhancing production and secretion of HBsAg/SVPs, as recently demonstrated [5]. The ability of the M protein to rescue SVP secretion may also contribute to this effect in HBV-infected, as opposed to transfected cells, a hypothesis which deserves future investigation in more efficient infectivity models. The low HBV infection rate in HepaRG cells hampers the monitoring of the fate of individual envelope proteins in infected cells. However, since S is the major component of the HBsAg and L is crucial for formation of enveloped virions, and both viral markers increased in HBV-infected HepaRG^EDEM3^ cells (Fig. 5A, B), it is conceivable to assume that neither envelope protein is significantly degraded in these cells.

Here we propose that EDEM3 overexpression is initially part of the UPR response to an altered protein homeostasis triggered by tumorigenesis, viral infection or other cellular disorders. By enhancing the proteasomal degradation, EDEM3 removes the burden of unwanted proteins, eventually alleviating the ER stress and preventing the cells from activating pro**-**apoptotic mechanisms. This is in agreement with very recent data showing that EDEM1, another member of the EDEM family, reduces the ER stress in pancreatic β**-**cells, by suppressing the IRE1**-** X**-**Box Binding Protein 1 (XBP1) pathway, which results in increased insulin production and secretion [25]. Concomitant upregulation of other ERAD factors in EDEM3-overexpressing cells, such as the E3 ubiquitin-protein ligase Hrd1, may be a contributing mechanism to the observed downregulation of the Rheb/mTOR pathway and hence activation of autophagy and increased HBV replication.

Surviving under chronic ER stress is a hallmark of highly**-**aggressive cancers [76]. Indeed, our data suggest that the elevated EDEM3 expression in tumor tissues could be part of such an adaptive mechanism. This hypothesis is supported by recent studies indicating high EDEM3 levels in prostate cancer patients, conferring cyto-protection against ER stressors [77]. Notably, EDEM3 overexpression induces resistance to radiotherapy in prostate cancer cells, while EDEM3 depletion triggers strong ER stress and restores cell sensitivity to treatment [77]. Similarly, analysis of the Human Protein Atlas (HPA) [78] reveals unfavourable prognoses in renal cancers overexpressing EDEM2 and EDEM3 [79]. Our analysis of HCC tumors also indicated a negative correlation between EDEM3 expression and patient survival, although this was not an independent prognostic factor. However, EDEM3 levels were significantly higher in patients resistant to sorafenib treatment, promoting this protein as a potential new target for anti-cancer therapy. Interestingly, the broad-spectrum mannosidase inhibitor, DMJ, was also shown to trigger ER stress and apoptosis in HCC cells [80]. It is tempting to hypothesize that the development of EDEM-specific inhibitors could aid in both antiviral and anticancer therapies; however, validation of such a therapeutic strategy will first require a better understanding of the ERAD functions in healthy and disordered tissues, by using relevant disease models and more clinical samples. Moreover, concomitant targeting of other host proteins with key roles in cell survival, such as the cellular inhibitor of apoptosis protein 2 (cIAP2) that has been involved in HBV-induced sorafenib resistance in liver cancer cells [81], may result in synergistic or potentiation effects and thus increase efficacy of cancer treatment.

## Conclusions

Our study indicates major implications of early ERAD factors in HBV infection and HCC development and progression that were previously unknown. By flagging the glycoproteins destined for degradation, EDEM3 increases the proteasomal flux and alleviates the UPR, protecting HBV-infected and tumor cells from ER stress-induced apoptosis. EDEM3 overexpression results in downregulation of the Rheb/mTOR pathway and enhanced secretory autophagy supporting HBV infection. Similar to other types of tumors, increased EDEM3 levels in HCC patients is associated with poor survival prognosis and resistance to therapy. Our work suggests that the ERAD gene expression pattern could be considered a prognostic factor in HCC development and promotes the ERAD pathway as an attractive target for the development of more effective antiviral and anticancer inhibitors.

## Supplementary Information

Additional file 1.

## Data Availability

All data generated or analysed during this study are included in this published article and its supplementary information files. Raw data are available from the corresponding authors on reasonable request.

## Abbreviations

ApoE: Apolipoprotein E
AKT: Protein kinase B
ATF6: Activating transcription factor 6
ATG3: Autophagy-related gene 3
BACE: Beta**-**site APP-cleaving enzyme 1
BAX: Pro-apoptotic effector B-cell lymphoma protein 2 (Bcl-2) Associated X
BiP: Binding immunoglobulin protein
BSA: Bovine serum albumin
Cas9: CRISPR associated protein 9
CHX: Cycloheximide
cIAP2: Cellular inhibitor of apoptosis protein 2
CPTAC: Clinical Proteomic Tumor Analysis Consortium
CRISPR: Clustered regularly interspaced palindromic repeats
CQ: Chloroquine
DAPI: 4′6-Diamidino-2-phenylindole
DMJ: Deoxymannojirimycine
dpi: Days post-infection
ECL: Enhanced chemiluminescence
EDEM: Endoplasmic reticulum degradation-enhancing α-mannosidase-like protein
EDTA: Ethylenediaminetetraacetic acid
ELISA: Enzyme-linked immunosorbent assay
eIF2α: α Subunit of eukaryotic initiation factor 2
ER: Endoplasmic reticulum
ERAD: Endoplasmic reticulum-associated degradation
FBS: Fetal bovine serum
FITC: Fluorescein isothiocyanate
GAPDH: Glyceraldehyde 3-phosphate dehydrogenase
GEO: Gene Expression Omnibus
GRP94: Glucose-regulated protein 94
HBsAg: Hepatitis B surface antigen
HBSS: Hank’s Balanced Salt Solution
HBV: Hepatitis B virus
HCC: Hepatocellular carcinoma
HDR: Homology-directed repair
HPA: Human Protein Atlas
Hrd1: HMG-CoA reductase degradation protein, Synoviolin
HRP: Horseradish peroxidase
HSC: Hematopoietic stem cells
IRE1α: Inositol-requiring enzyme 1α
Kif: Kifunensine
L: Large
LC3: Microtubule-associated protein light chain 3
LIHC: Liver Hepatocellular Carcinoma
M: Medium
mTOR: Mammalian target of rapamycin
MVBs: Multivesicular bodies
N: Normal tissue
NAT: Normal adjacent to tumor tissue
NTCP: Sodium taurocholate co-transporting polypeptide
OS-9: Osteosarcoma amplified-9
PBS: Phosphate buffered saline
PCR: Polymerase chain reaction
PDI: Protein disulfide isomerase
PERK: Double-stranded RNA-activated protein kinase (PKR)-like ER kinase
PFA: Paraformaldehyde
PHH: Primary human hepatocyte
PI3K: Phosphatidylinositol 3-kinase
PTEN: Phosphase and tensin human
Rheb: Ras homolog enriched in the brain
RT: Reverse-transcription
S: Small
Sel1L: Suppressor of Lin-12-like
SD: Standard deviations
SVPs: Subviral particles
T: Tumor tissue
TCGA: The Cancer Genome Atlas
TBP: TATA-Box Binding Protein
TM: Tunicamycin
TyrST: Soluble Tyrosinase
UALCAN: University of Alabama at Birmingham Cancer data analysis Portal
UPR: Unfolded protein response
WNT5A: Wingless-type family member 5A
XBP1: IRE1-X-Box Binding Protein 1
XTP3-B: ER lectin 1, ERLEC1

## References

[1] Gnyawali B, Pusateri A, Nickerson A, Jalil S, Mumtaz K. Epidemiologic and socioeconomic factors impacting hepatitis B virus and related hepatocellular carcinoma. World J Gastroenterol. 2022;28(29):3793–802. 36157533 10.3748/wjg.v28.i29.3793 PMC9367226

[2] Llovet JM, Kelley RK, Villanueva A, Singal AG, Pikarsky E, Roayaie S, Lencioni R, Koike K, Zucman-Rossi J, Finn RS. Hepatocellular carcinoma. Nat Rev Dis Primers. 2021;7(1):6. 33479224 10.1038/s41572-020-00240-3 PMC13537433

[3] Li J, Lin Y, Wang X, Lu M. Interconnection of cellular autophagy and endosomal vesicle trafficking and its role in hepatitis B virus replication and release. Virol Sin. 2024;39(1):24–30. 38211880 10.1016/j.virs.2024.01.001 PMC10877419

[4] Prange R. Host factors involved in hepatitis B virus maturation, assembly, and egress. Med Microbiol Immunol. 2012;201(4):449–61. 22965171 10.1007/s00430-012-0267-9

[5] Wang X, Wei Z, Cheng B, Li J, He Y, Lan T, Kemper T, Lin Y, Jiang B, Jiang Y, Meng Z, Lu M. Endoplasmic reticulum stress promotes HBV production by enhancing use of the autophagosome/multivesicular body axis. Hepatology. 2022;75(2):438–54. 34580902 10.1002/hep.32178

[6] Liang Y, Luo X, Schefczyk S, Muungani LT, Deng H, Wang B, Baba HA, Lu M, Wedemeyer H, Schmidt HH, Broering R. Hepatitis B surface antigen expression impairs endoplasmic reticulum stress-related autophagic flux by decreasing LAMP2. JHEP Rep. 2024;6(4): 101012. 38425451 10.1016/j.jhepr.2024.101012 PMC10899050

[7] Bertolotti A, Zhang Y, Hendershot L, Harding HP, Ron D. Dynamic interaction of BiP and ER stress transducers in the unfolded-protein response. Nat Cell Biol. 2000;2(6):326–32. 10854322 10.1038/35014014

[8] Walter P, Ron D. The unfolded protein response: from stress pathway to homeostatic regulation. Science. 2011;334(6059):1081–6. 22116877 10.1126/science.1209038

[9] Hoseki J, Ushioda R, Nagata K. Mechanism and components of endoplasmic reticulum-associated degradation. J Biochem. 2010;147(1):19–25. 19923195 10.1093/jb/mvp194

[10] Hwang J, Qi L. Quality control in the endoplasmic reticulum: crosstalk between ERAD and UPR pathways. Trends Biochem Sci. 2018;43(8):593–605. 30056836 10.1016/j.tibs.2018.06.005 PMC6327314

[11] Molinari M, Calanca V, Galli C, Lucca P, Paganetti P. Role of EDEM in the release of misfolded glycoproteins from the calnexin cycle. Science. 2003;299(5611):1397–400. 12610306 10.1126/science.1079474

[12] Ninagawa S, Okada T, Sumitomo Y, Kamiya Y, Kato K, Horimoto S, Ishikawa T, Takeda S, Sakuma T, Yamamoto T, Mori K. EDEM2 initiates mammalian glycoprotein ERAD by catalyzing the first mannose trimming step. J Cell Biol. 2014;206(3):347–56. 25092655 10.1083/jcb.201404075 PMC4121980

[13] Oda Y, Hosokawa N, Wada I, Nagata K. EDEM as an acceptor of terminally misfolded glycoproteins released from calnexin. Science. 2003;299(5611):1394–7. 12610305 10.1126/science.1079181

[14] Lazar C, Macovei A, Petrescu S, Branza-Nichita N. Activation of ERAD pathway by human hepatitis B virus modulates viral and subviral particle production. PLoS ONE. 2012;7(3):e34169. 22461906 10.1371/journal.pone.0034169 PMC3312915

[15] Lazar C, Uta M, Petrescu SM, Branza-Nichita N. Novel function of the endoplasmic reticulum degradation-enhancing alpha-mannosidase-like proteins in the human hepatitis B virus life cycle, mediated by the middle envelope protein. Cell Microbiol. 2017. 10.1111/cmi.12653. 27490136 10.1111/cmi.12653

[16] Macovei A, Petrareanu C, Lazar C, Florian P, Branza-Nichita N. Regulation of hepatitis B virus infection by Rab5, Rab7, and the endolysosomal compartment. J Virol. 2013;87(11):6415–27. 23536683 10.1128/JVI.00393-13 PMC3648082

[17] Manica G, Ghenea S, Munteanu CVA, Martin EC, Butnaru C, Surleac M, Chiritoiu GN, Alexandru PR, Petrescu AJ, Petrescu SM. EDEM3 domains cooperate to perform its overall cell functioning. Int J Mol Sci. 2021;22(4):2172. 33671632 10.3390/ijms22042172 PMC7926307

[18] Munteanu CVA, Chiritoiu GN, Chiritoiu M, Ghenea S, Petrescu AJ, Petrescu SM. Affinity proteomics and deglycoproteomics uncover novel EDEM2 endogenous substrates and an integrative ERAD network. Mol Cell Proteomics. 2021;20: 100125. 34332121 10.1016/j.mcpro.2021.100125 PMC8455867

[19] Lazar C, Durantel D, Macovei A, Zitzmann N, Zoulim F, Dwek RA, Branza-Nichita N. Treatment of hepatitis B virus-infected cells with alpha-glucosidase inhibitors results in production of virions with altered molecular composition and infectivity. Antiviral Res. 2007;76(1):30–7. 17548120 10.1016/j.antiviral.2007.04.004

[20] Vanoni O, Paganetti P, Molinari M. Consequences of individual N-glycan deletions and of proteasomal inhibition on secretion of active BACE. Mol Biol Cell. 2008;19(10):4086–98. 18632981 10.1091/mbc.E08-05-0459 PMC2555951

[21] Popescu CI, Paduraru C, Dwek RA, Petrescu SM. Soluble tyrosinase is an endoplasmic reticulum (ER)-associated degradation substrate retained in the ER by calreticulin and BiP/GRP78 and not calnexin. J Biol Chem. 2005;280(14):13833–40. 15677452 10.1074/jbc.M413087200

[22] Macovei A, Radulescu C, Lazar C, Petrescu S, Durantel D, Dwek RA, Zitzmann N, Nichita NB. Hepatitis B virus requires intact caveolin-1 function for productive infection in HepaRG cells. J Virol. 2010;84(1):243–53. 19846513 10.1128/JVI.01207-09 PMC2798426

[23] Livak KJ, Schmittgen TD. Analysis of relative gene expression data using real-time quantitative PCR and the 2(-Delta Delta C(T)) Method. Methods. 2001;25(4):402–8. 11846609 10.1006/meth.2001.1262

[24] Schmittgen TD, Livak KJ. Analyzing real-time PCR data by the comparative C(T) method. Nat Protoc. 2008;3(6):1101–8. 18546601 10.1038/nprot.2008.73

[25] Flintoaca Alexandru PR, Chiritoiu GN, Lixandru D, Zurac S, Ionescu-Targoviste C, Petrescu SM. EDEM1 regulates the insulin mRNA level by inhibiting the endoplasmic reticulum stress-induced IRE1/JNK/c-Jun pathway. iScience. 2023;26(10):107956. 37822496 10.1016/j.isci.2023.107956 PMC10562789

[26] Davis S, Meltzer PS. GEOquery: a bridge between the gene expression omnibus (GEO) and BioConductor. Bioinformatics. 2007;23(14):1846–7. 17496320 10.1093/bioinformatics/btm254

[27] Colaprico A, Silva TC, Olsen C, Garofano L, Cava C, Garolini D, Sabedot TS, Malta TM, Pagnotta SM, Castiglioni I, Ceccarelli M, Bontempi G, Noushmehr H. TCGAbiolinks: an R/Bioconductor package for integrative analysis of TCGA data. Nucleic Acids Res. 2016;44(8): e71. 26704973 10.1093/nar/gkv1507 PMC4856967

[28] Goldman MJ, Craft B, Hastie M, Repecka K, McDade F, Kamath A, Banerjee A, Luo Y, Rogers D, Brooks AN, Zhu J, Haussler D. Visualizing and interpreting cancer genomics data via the Xena platform. Nat Biotechnol. 2020;38(6):675–8. 32444850 10.1038/s41587-020-0546-8 PMC7386072

[29] Chandrashekar DS, Bashel B, Balasubramanya SAH, Creighton CJ, Ponce-Rodriguez I, Chakravarthi B, Varambally S. UALCAN: a portal for facilitating tumor subgroup gene expression and survival analyses. Neoplasia. 2017;19(8):649–58. 28732212 10.1016/j.neo.2017.05.002 PMC5516091

[30] Chandrashekar DS, Karthikeyan SK, Korla PK, Patel H, Shovon AR, Athar M, Netto GJ, Qin ZS, Kumar S, Manne U, Creighton CJ, Varambally S. UALCAN: An update to the integrated cancer data analysis platform. Neoplasia. 2022;25:18–27. 35078134 10.1016/j.neo.2022.01.001 PMC8788199

[31] Pinyol R, Montal R, Bassaganyas L, Sia D, Takayama T, Chau GY, Mazzaferro V, Roayaie S, Lee HC, Kokudo N, Zhang Z, Torrecilla S, Moeini A, Rodriguez-Carunchio L, Gane E, Verslype C, Croitoru AE, Cillo U, de la Mata M, Lupo L, Strasser S, Park JW, Camps J, Sole M, Thung SN, Villanueva A, Pena C, Meinhardt G, Bruix J, Llovet JM. Molecular predictors of prevention of recurrence in HCC with sorafenib as adjuvant treatment and prognostic factors in the phase 3 STORM trial. Gut. 2019;68(6):1065–75. 30108162 10.1136/gutjnl-2018-316408 PMC6580745

[32] Zhang J, Zheng B, Zhou X, Zheng T, Wang H, Wang Y, Zhang W. Increased BST-2 expression by HBV infection promotes HBV-associated HCC tumorigenesis. J Gastrointest Oncol. 2021;12(2):694–710. 34012659 10.21037/jgo-20-356 PMC8107608

[33] Gripon P, Rumin S, Urban S, Le Seyec J, Glaise D, Cannie I, Guyomard C, Lucas J, Trepo C, Guguen-Guillouzo C. Infection of a human hepatoma cell line by hepatitis B virus. Proc Natl Acad Sci U S A. 2002;99(24):15655–60. 12432097 10.1073/pnas.232137699 PMC137772

[34] Sun Y, Qi Y, Peng B, Li W. NTCP-reconstituted in vitro HBV infection system. Methods Mol Biol. 2017;1540:1–14. 27975303 10.1007/978-1-4939-6700-1_1

[35] Arbuthnot P, Kew M. Hepatitis B virus and hepatocellular carcinoma. Int J Exp Pathol. 2001;82(2):77–100. 11454100 10.1111/j.1365-2613.2001.iep0082-0077-x PMC2517704

[36] Chuang YC, James Ou JH. Regulation of hepatitis B virus replication by autophagic membranes. Autophagy. 2023;19(4):1357–8. 36037301 10.1080/15548627.2022.2117974 PMC10012931

[37] George G, Ninagawa S, Yagi H, Furukawa JI, Hashii N, Ishii-Watabe A, Deng Y, Matsushita K, Ishikawa T, Mamahit YP, Maki Y, Kajihara Y, Kato K, Okada T, Mori K. Purified EDEM3 or EDEM1 alone produces determinant oligosaccharide structures from M8B in mammalian glycoprotein ERAD. Elife. 2021;10: e70357. 34698634 10.7554/eLife.70357 PMC8570694

[38] Malhotra JD, Kaufman RJ. The endoplasmic reticulum and the unfolded protein response. Semin Cell Dev Biol. 2007;18(6):716–31. 18023214 10.1016/j.semcdb.2007.09.003 PMC2706143

[39] Mueller B, Lilley BN, Ploegh HL. SEL1L, the homologue of yeast Hrd3p, is involved in protein dislocation from the mammalian ER. J Cell Biol. 2006;175(2):261–70. 17043138 10.1083/jcb.200605196 PMC2064567

[40] Christianson JC, Shaler TA, Tyler RE, Kopito RR. OS-9 and GRP94 deliver mutant alpha1-antitrypsin to the Hrd1-SEL1L ubiquitin ligase complex for ERAD. Nat Cell Biol. 2008;10(3):272–82. 18264092 10.1038/ncb1689 PMC2757077

[41] Lin LL, Wang HH, Pederson B, Wei X, Torres M, Lu Y, Li ZJ, Liu X, Mao H, Wang H, Zhou LE, Zhao Z, Sun S, Qi L. SEL1L-HRD1 interaction is required to form a functional HRD1 ERAD complex. Nat Commun. 2024;15(1):1440. 38365914 10.1038/s41467-024-45633-0 PMC10873344

[42] Mizushima N, Yoshimori T. How to interpret LC3 immunoblotting. Autophagy. 2007;3(6):542–5. 17611390 10.4161/auto.4600

[43] Tanida I, Ueno T, Kominami E. LC3 and autophagy. Methods Mol Biol. 2008;445:77–88. 18425443 10.1007/978-1-59745-157-4_4

[44] Mauthe M, Orhon I, Rocchi C, Zhou X, Luhr M, Hijlkema KJ, Coppes RP, Engedal N, Mari M, Reggiori F. Chloroquine inhibits autophagic flux by decreasing autophagosome-lysosome fusion. Autophagy. 2018;14(8):1435–55. 29940786 10.1080/15548627.2018.1474314 PMC6103682

[45] Wu J, Chen S, Liu H, Zhang Z, Ni Z, Chen J, Yang Z, Nie Y, Fan D. Tunicamycin specifically aggravates ER stress and overcomes chemoresistance in multidrug-resistant gastric cancer cells by inhibiting N-glycosylation. J Exp Clin Cancer Res. 2018;37(1):272. 30413206 10.1186/s13046-018-0935-8 PMC6230241

[46] Saxton RA, Sabatini DM. mTOR signaling in growth, metabolism, and disease. Cell. 2017;168(6):960–76. 28283069 10.1016/j.cell.2017.02.004 PMC5394987

[47] Rabanal-Ruiz Y, Otten EG, Korolchuk VI. mTORC1 as the main gateway to autophagy. Essays Biochem. 2017;61(6):565–84. 29233869 10.1042/EBC20170027 PMC5869864

[48] Sato T, Umetsu A, Tamanoi F. Characterization of the Rheb-mTOR signaling pathway in mammalian cells: constitutive active mutants of Rheb and mTOR. Methods Enzymol. 2008;438:307–20. 18413257 10.1016/S0076-6879(07)38021-X PMC2693245

[49] Carroll B, Maetzel D, Maddocks OD, Otten G, Ratcliff M, Smith GR, Dunlop EA, Passos JF, Davies OR, Jaenisch R, Tee AR, Sarkar S, Korolchuk VI. Control of TSC2-Rheb signaling axis by arginine regulates mTORC1 activity. Elife. 2016;5: e11058. 26742086 10.7554/eLife.11058 PMC4764560

[50] Liu L, Inoki A, Fan K, Mao F, Shi G, Jin X, Zhao M, Ney G, Jones M, Sun S, Dou Y, Inoki K, Qi L, Li Q. ER-associated degradation preserves hematopoietic stem cell quiescence and self-renewal by restricting mTOR activity. Blood. 2020;136(26):2975–86. 33150381 10.1182/blood.2020007975 PMC7770563

[51] Fote GM, Geller NR, Efstathiou NE, Hendricks N, Vavvas DG, Reidling JC, Thompson LM, Steffan JS. Isoform-dependent lysosomal degradation and internalization of apolipoprotein E requires autophagy proteins. J Cell Sci. 2022;135(2):jcs258687. 34982109 10.1242/jcs.258687 PMC8917355

[52] Zhang J, Guo J, Yang N, Huang Y, Hu T, Rao C. Endoplasmic reticulum stress-mediated cell death in liver injury. Cell Death Dis. 2022;13(12):1051. 36535923 10.1038/s41419-022-05444-x PMC9763476

[53] Malhi H, Kaufman RJ. Endoplasmic reticulum stress in liver disease. J Hepatol. 2011;54(4):795–809. 21145844 10.1016/j.jhep.2010.11.005 PMC3375108

[54] Liu T, Zhu W, Yang X, Chen L, Yang R, Hua Z, Li G. Detection of apoptosis based on the interaction between annexin V and phosphatidylserine. Anal Chem. 2009;81(6):2410–3. 19219984 10.1021/ac801267s

[55] Chipuk JE, Kuwana T, Bouchier-Hayes L, Droin NM, Newmeyer DD, Schuler M, Green DR. Direct activation of Bax by p53 mediates mitochondrial membrane permeabilization and apoptosis. Science. 2004;303(5660):1010–4. 14963330 10.1126/science.1092734

[56] Chu JYK, Chuang YC, Tsai KN, Pantuso J, Ishida Y, Saito T, Ou JJ. Autophagic membranes participate in hepatitis B virus nucleocapsid assembly, precore and core protein trafficking, and viral release. Proc Natl Acad Sci U S A. 2022;119(30): e2201927119. 35858426 10.1073/pnas.2201927119 PMC9335259

[57] Wang X, Wei Z, Jiang Y, Meng Z, Lu M. mTOR signaling: the interface linking cellular metabolism and hepatitis B virus replication. Virol Sin. 2021;36(6):1303–14. 34580816 10.1007/s12250-021-00450-3 PMC8692646

[58] Xiang K, Wang B. Role of the PI3K-AKT-mTOR pathway in hepatitis B virus infection and replication. Mol Med Rep. 2018;17(3):4713–9. 29328380 10.3892/mmr.2018.8395

[59] Ni Y, Lempp FA, Mehrle S, Nkongolo S, Kaufman C, Falth M, Stindt J, Koniger C, Nassal M, Kubitz R, Sultmann H, Urban S. Hepatitis B and D viruses exploit sodium taurocholate co-transporting polypeptide for species-specific entry into hepatocytes. Gastroenterology. 2014;146(4):1070–83. 24361467 10.1053/j.gastro.2013.12.024

[60] Elbein AD, Tropea JE, Mitchell M, Kaushal GP. Kifunensine, a potent inhibitor of the glycoprotein processing mannosidase I. J Biol Chem. 1990;265(26):15599–605. 2144287

[61] Fuhrmann U, Bause E, Legler G, Ploegh H. Novel mannosidase inhibitor blocking conversion of high mannose to complex oligosaccharides. Nature. 1984;307(5953):755–8. 6230538 10.1038/307755a0

[62] Martins-Filho SN, Paiva C, Azevedo RS, Alves VAF. Histological grading of hepatocellular carcinoma-a systematic review of literature. Front Med (Lausanne). 2017;4:193. 29209611 10.3389/fmed.2017.00193 PMC5701623

[63] Duseja A. Staging of hepatocellular carcinoma. J Clin Exp Hepatol. 2014;4(Suppl 3):S74-79. 25755615 10.1016/j.jceh.2014.03.045 PMC4284240

[64] Mazzoccoli G, Miele L, Oben J, Grieco A, Vinciguerra M. Biology, epidemiology, clinical aspects of hepatocellular carcinoma and the role of sorafenib. Curr Drug Targets. 2016;17(7):783–99. 26648069 10.2174/1389450117666151209120831

[65] Zhang K, Zhang Q, Jia R, Xiang S, Xu L. A comprehensive review of the relationship between autophagy and sorafenib-resistance in hepatocellular carcinoma: ferroptosis is noteworthy. Front Cell Dev Biol. 2023;11:1156383. 37181755 10.3389/fcell.2023.1156383 PMC10172583

[66] Garten A, Grohmann T, Kluckova K, Lavery GG, Kiess W, Penke M. Sorafenib-induced apoptosis in hepatocellular carcinoma is reversed by SIRT1. Int J Mol Sci. 2019;20(16):4048. 31430957 10.3390/ijms20164048 PMC6719220

[67] Chen X, Cubillos-Ruiz JR. Endoplasmic reticulum stress signals in the tumour and its microenvironment. Nat Rev Cancer. 2021;21(2):71–88. 33214692 10.1038/s41568-020-00312-2 PMC7927882

[68] Oakes SA. Endoplasmic reticulum stress signaling in cancer cells. Am J Pathol. 2020;190(5):934–46. 32112719 10.1016/j.ajpath.2020.01.010 PMC7237829

[69] Marciniak SJ, Chambers JE, Ron D. Pharmacological targeting of endoplasmic reticulum stress in disease. Nat Rev Drug Discov. 2022;21(2):115–40. 34702991 10.1038/s41573-021-00320-3

[70] Bonsignore G, Martinotti S, Ranzato E. Endoplasmic reticulum stress and cancer: could unfolded protein response be a druggable target for cancer therapy? Int J Mol Sci. 2023;24(2):1566. 36675080 10.3390/ijms24021566 PMC9865308

[71] Tan X, He X, Fan Z. Upregulation of HRD1 promotes cell migration and invasion in colon cancer. Mol Cell Biochem. 2019;454(1–2):1–9. 30306455 10.1007/s11010-018-3447-0

[72] Ji F, Zhou M, Sun Z, Jiang Z, Zhu H, Xie Z, Ouyang X, Zhang L, Li L. Integrative proteomics reveals the role of E3 ubiquitin ligase SYVN1 in hepatocellular carcinoma metastasis. Cancer Commun (Lond). 2021;41(10):1007–23. 34196494 10.1002/cac2.12192 PMC8504139

[73] Liu L, Long H, Wu Y, Li H, Dong L, Zhong JL, Liu Z, Yang X, Dai X, Shi L, Ren M, Lin Z. HRD1-mediated PTEN degradation promotes cell proliferation and hepatocellular carcinoma progression. Cell Signal. 2018;50:90–9. 29958993 10.1016/j.cellsig.2018.06.011

[74] Bhattacharya A, Wei J, Song W, Gao B, Tian C, Wu SA, Wang J, Chen L, Fang D, Qi L. SEL1L-HRD1 ER-associated degradation suppresses hepatocyte hyperproliferation and liver cancer. iScience. 2022;25(10):105183. 36238898 10.1016/j.isci.2022.105183 PMC9550610

[75] Lin Y, Zhao Z, Huang A, Lu M. Interplay between cellular autophagy and hepatitis b virus replication: a systematic review. Cells. 2020;9(9):2101. 32942717 10.3390/cells9092101 PMC7563265

[76] Chen X, Iliopoulos D, Zhang Q, Tang Q, Greenblatt MB, Hatziapostolou M, Lim E, Tam WL, Ni M, Chen Y, Mai J, Shen H, Hu DZ, Adoro S, Hu B, Song M, Tan C, Landis MD, Ferrari M, Shin SJ, Brown M, Chang JC, Liu XS, Glimcher LH. XBP1 promotes triple-negative breast cancer by controlling the HIF1alpha pathway. Nature. 2014;508(7494):103–7. 24670641 10.1038/nature13119 PMC4105133

[77] Scott E, Garnham R, Cheung K, Duxfield A, Elliott DJ, Munkley J. Pro-survival factor EDEM3 confers therapy resistance in prostate cancer. Int J Mol Sci. 2022;23(15):8184. 35897761 10.3390/ijms23158184 PMC9332126

[78] Uhlen M, Fagerberg L, Hallstrom BM, Lindskog C, Oksvold P, Mardinoglu A, Sivertsson A, Kampf C, Sjostedt E, Asplund A, Olsson I, Edlund K, Lundberg E, Navani S, Szigyarto CA, Odeberg J, Djureinovic D, Takanen JO, Hober S, Alm T, Edqvist PH, Berling H, Tegel H, Mulder J, Rockberg J, Nilsson P, Schwenk JM, Hamsten M, von Feilitzen K, Forsberg M, Persson L, Johansson F, Zwahlen M, von Heijne G, Nielsen J, Ponten F. Proteomics. Tissue-based map of the human proteome. Science. 2015;347(6220):1260419. 25613900 10.1126/science.1260419

[79] Tax G, Lia A, Santino A, Roversi P. Modulation of ERQC and ERAD: a broad-spectrum spanner in the works of cancer cells? J Oncol. 2019;2019:8384913. 31662755 10.1155/2019/8384913 PMC6791201

[80] Lu Y, Xu YY, Fan KY, Shen ZH. 1-Deoxymannojirimycin, the alpha1,2-mannosidase inhibitor, induced cellular endoplasmic reticulum stress in human hepatocarcinoma cell 7721. Biochem Biophys Res Commun. 2006;344(1):221–5. 16615997 10.1016/j.bbrc.2006.03.111

[81] Zhang S, Li N, Sheng Y, Chen W, Ma Q, Yu X, Lian J, Zeng J, Yang Y, Yan J. Hepatitis B virus induces sorafenib resistance in liver cancer via upregulation of cIAP2 expression. Infect Agent Cancer. 2021;16(1):20. 33757557 10.1186/s13027-021-00359-2 PMC7988944

