# Supplementary material for: The endoplasmic reticulum degradation-enhancing α-mannosidase-like protein 3 attenuates the unfolded protein response and has pro-survival and pro-viral roles in hepatoma cells and hepatocellular carcinoma patients
Source: J Biomed Sci. 2025 Jan 22;32:11. doi: 10.1186/s12929-024-01103-9 (PMC11752926; doi:10.1186/s12929-024-01103-9)
Supplement: Supplementary file 1 — Additional file 1. [file 12929_2024_1103_MOESM1_ESM.docx]

**Additional file for**

**The endoplasmic reticulum degradation-enhancing α-mannosidase-like protein 3 attenuates the unfolded protein response and has pro-survival and pro-viral roles in hepatoma cells and hepatocellular carcinoma patients**

Alina-Veronica Ghionescu^1,2*^, Mihaela Uta^1,2*^, Andrei Sorop^2^, Catalin Lazar^1^, Petruta R. Flintoaca-Alexandru^3^, Gabriela Chiritoiu^3^, Livia Sima^3^, Stefana-Maria Petrescu^3^, Simona Olimpia Dima^2,4#^, Norica Branza-Nichita^1#^

^1^Institute of Biochemistry of the Romanian Academy, Department of Viral Glycoproteins, Bucharest, Romania

^2^Center of Excellence in Translational Medicine, Fundeni Clinical Institute, Bucharest, Romania

^3^Institute of Biochemistry of the Romanian Academy, Department of Molecular Cell Biology, Bucharest, Romania

^4^Digestive Diseases and Liver Transplantation Center, Fundeni Clinical Institute, Bucharest, Romania

^#^Correspondence to:

Norica Nichita, PhD, Institute of Biochemistry of the Romanian Academy, Splaiul Independentei 296, Sector 6 Bucharest, 060031, Romania; nichita@biochim.ro; Tel: (+4).021.223.90.69, Fax: (+4).021.223.90.69; https://orcid.org/0000-0001-7896-7930.

Simona Olimpia Dima, MD PhD, Center of Excellence in Translational Medicine, Fundeni Clinical Institute and Digestive Diseases and Liver Transplantation Center, Fundeni Clinical Institute, Soseaua Fundeni 258, Sector 2 Bucharest, 022328, Romania; [dima.simona@gmail.com](mailto:dima.simona@gmail.com), Tel: (+4).021.275.05.00; https://orcid.org/0000-0001-5275-8938

^*^Authors with equal contributions to this work


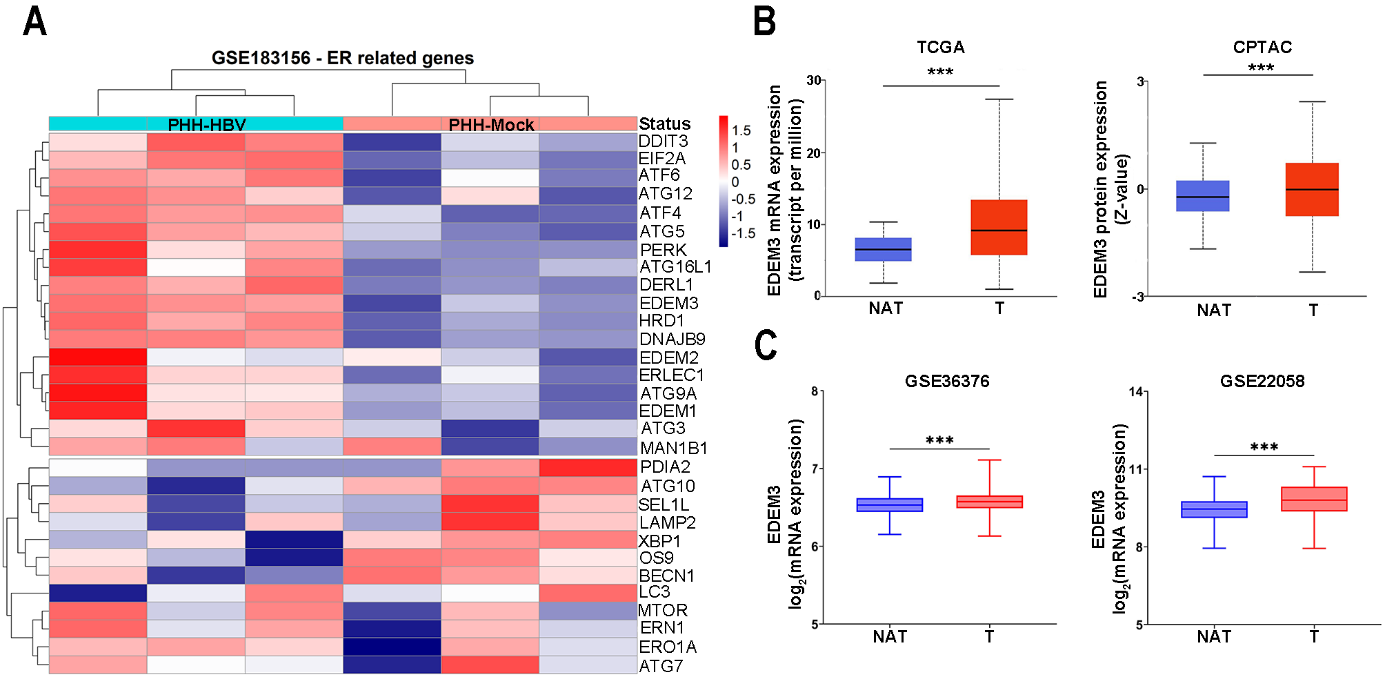


**Supplementary Figure 1.** EDEM3 increases in HBV-infected PHH and HCC tissues.

(**A**) Heatmap correlation of ER-associated genes profile in uninfected (PHH-Mock) and HBV-infected PHH (PHH-HBV).

(**B**) EDEM3 mRNA and protein levels in NAT and T tissues in the TCGA-LIHC (*n*=421, of which 50 NAT and 371 T) and the CPTAC cohorts (*n*=330, of which 165 NAT and 165 T). Data analysis was performed in the UALCAN database (**, *p*<0.01; ****, *p* < 0.0001).

(**C**) Boxplot depicting EDEM3 mRNA expression in NAT and T tissues across multiple HCC cohorts: GSE36376 (*n*=433, of which 193 NAT and 240 T) and GSE22058 (*n*=197, of which 97 NAT and 100 T). The data were analyzed using unpaired t-test (***, *p* < 0.001).


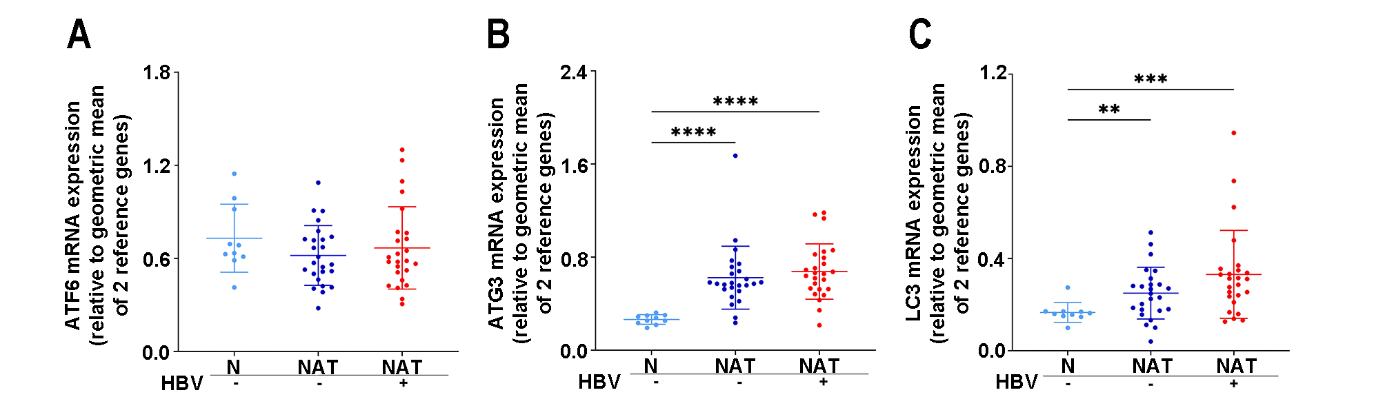


**Supplementary Figure 2.** Analysis of UPR and autophagy markers in HCC tissues.

ATF6 (**A**), ATG3 (**B**) and LC3 mRNA expression (**C**) was quantified in 10 N and 50 NAT from two equally distributed HCC patients cohorts, without (-) or with HBV infection (+), by RT real-time PCR, using a 2^−ΔCt^ formula. Data was normalized to GAPDH and TBP expression. Each scatter plot illustrates the median with the standard deviation of gene expression. Comparisons between groups were performed using unpaired t-test (**, *p* < 0.01; ***, *p* < 0.001; ****, *p* < 0.0001).


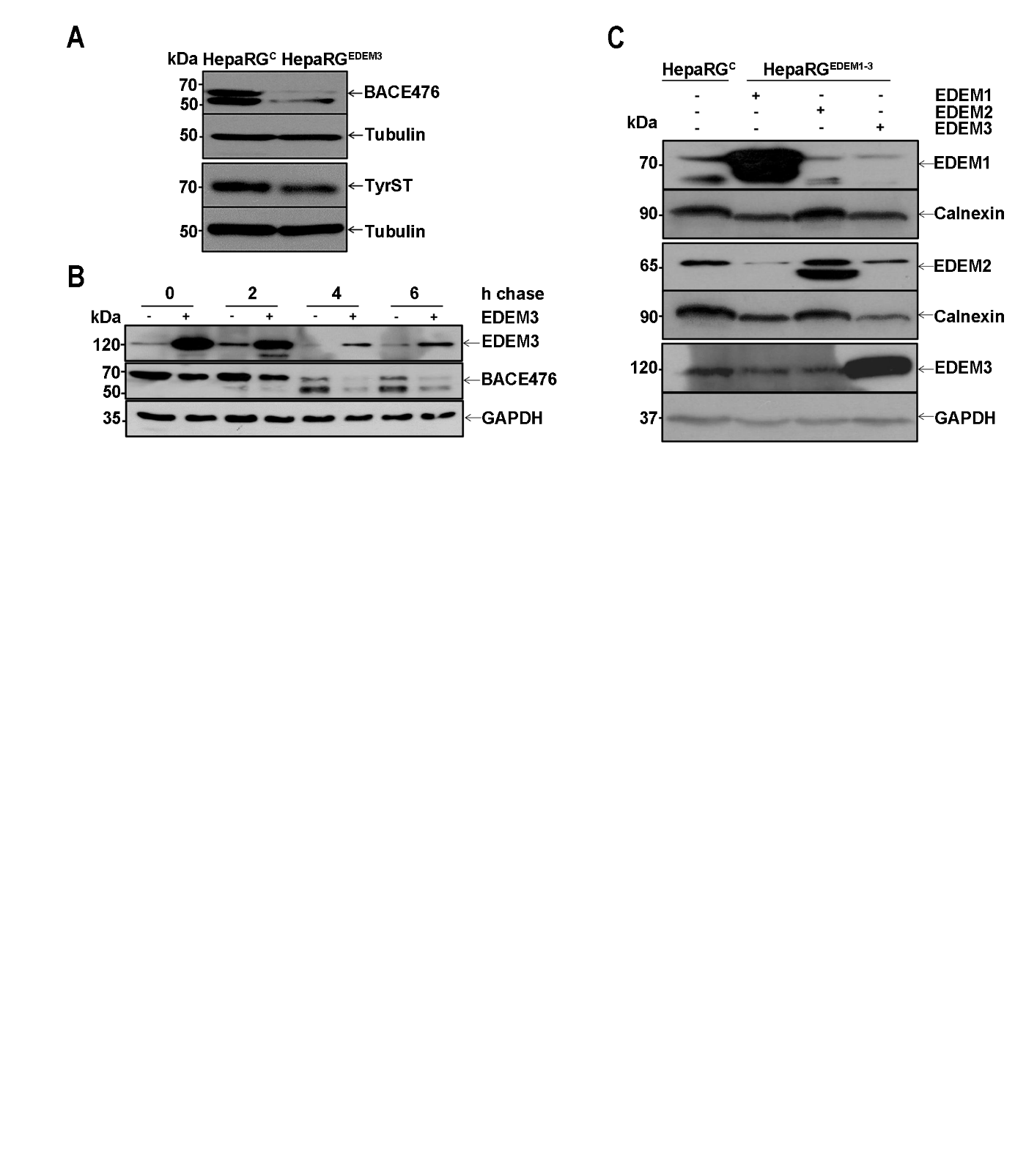


**Supplementary Figure 3.** Characterization of the EDEM3-overexpressing HepaRG cell line.

(**A**) Lysates of HepaRG^EDEM3^ and HepaRG^C^ cells transfected with plasmids encoding for BACE476 and TyrST were subjected to Western blot and detection with corresponding antibodies.

(**B**) HepaRG^C^ (-) and HepaRG^EDEM3^ (+) cells were transfected with the plasmid encoding for BACE476 and cultured for 48 h, before addition of CHX to stop the protein translation. At indicated time-points cells were collected and BACE476 was detected by Western blot with corresponding antibodies.

(**C**) EDEM1-3 expression was evaluated by Western blot in HepaRG^C^, HepaRG^EDEM1^, HepaRG^EDEM2^ and HepaRG^EDEM3^ cell lines. Calnexin or GAPDH detection was used as total protein loading control.


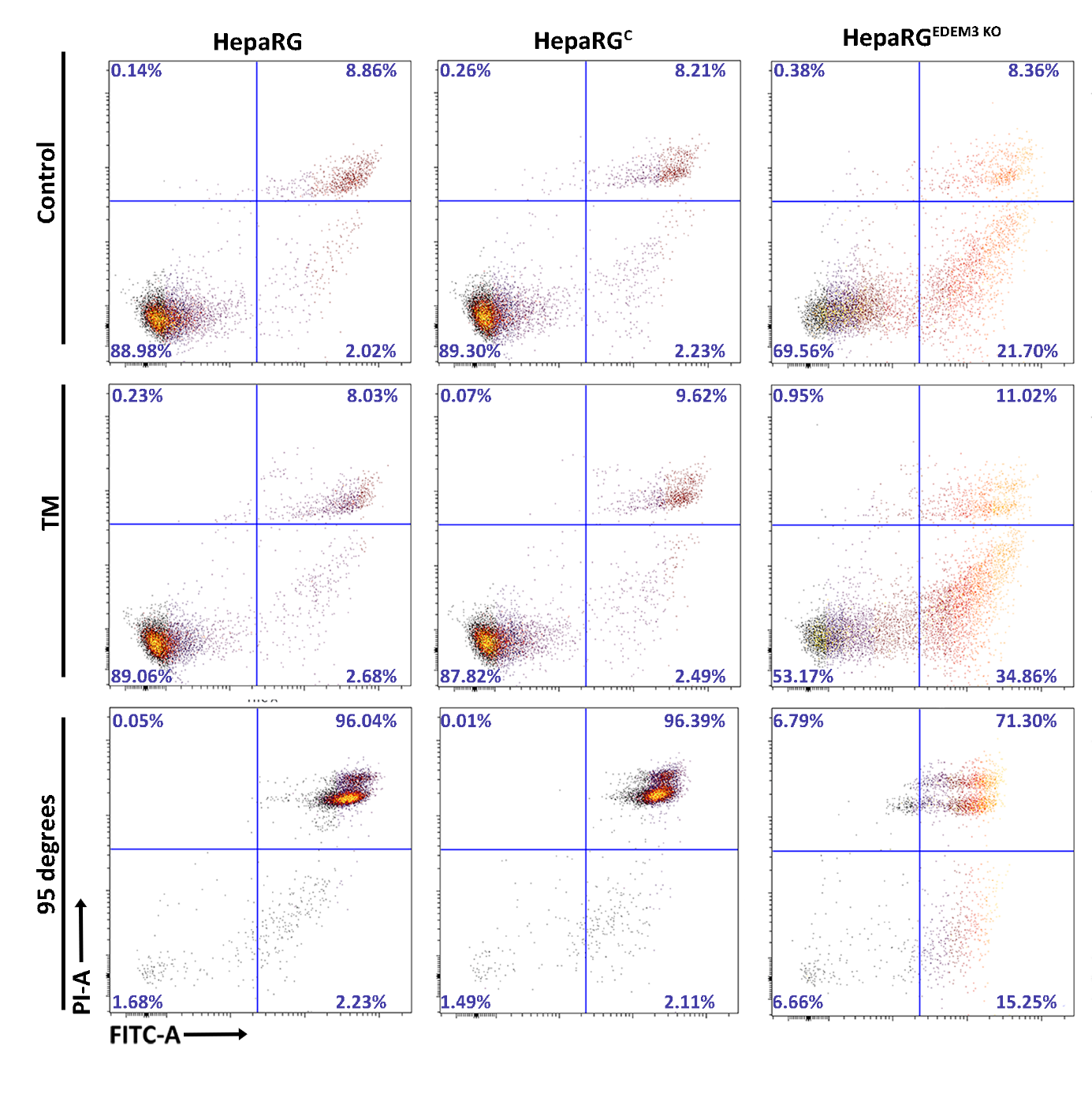


**Supplementary Figure 4.** EDEM3 depletion enhances apoptosis in HepaRG cells.

Cell apoptosis was evaluated by flow cytometry, using FITC-labelled [Annexin](https://www.sciencedirect.com/topics/biochemistry-genetics-and-molecular-biology/annexin) V and propidium iodide (PI) staining. Where indicated, chemical treatment (TM 2.5 µg/mL, for 6 h) and heat exposure (95^o^C, for 5 min) as control for induced apoptosis. Representative results of two independent experiments are shown.


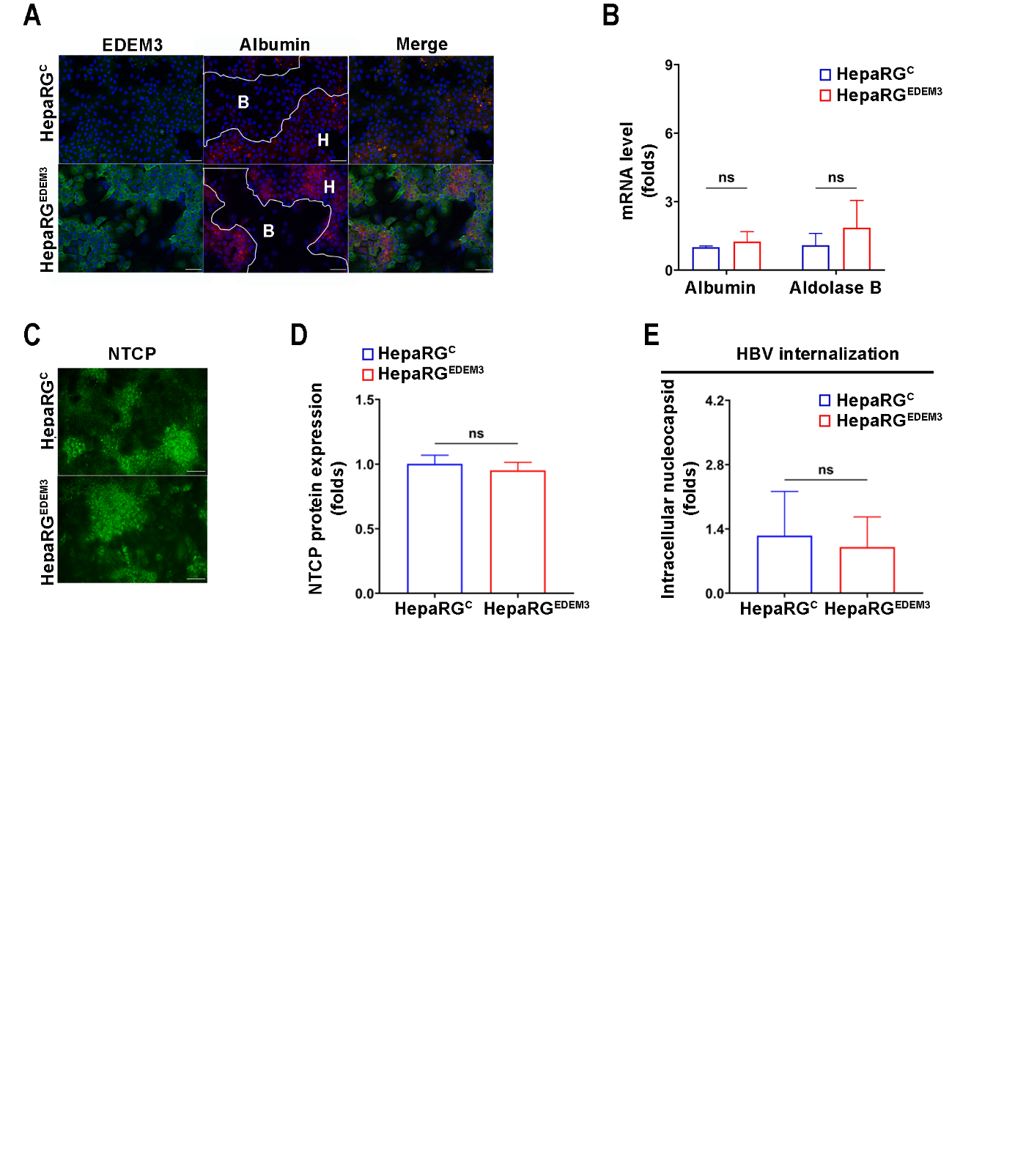


**Supplementary Figure 5.** Differentiation of the HepaRG cells with upregulated EDEM3 expression.

(**A**) HepaRG^EDEM3^ and HepaRG^C^ cells were subjected to differentiation followed by immunofluorescence microscopy to reveal EDEM3 expression (green) and the hepatocyte marker, albumin (red) with corresponding antibodies. Hepatocyte (H)- and biliary (B)-like cell populations are shown. The nuclei were stained with DAPI. Images were taken with a Zeiss AxioImager.Z1 inverted microscope and analyzed with AxioVision SE64 Rel. 4.9.1 Software. Scale bar is 50 μm.

(**B**) Quantification of albumin and aldolase B transcripts in HepaRG^EDEM3^ and HepaRG^C^ by RT real-time PCR. The results represent the data and standard deviations from two independent experiments. Statistical analysis was performed by using unpaired t-test; ns, non-statistical relevance.

(**C**) As in (**A**), except that NTCP expression was analyzed with appropriate antibodies. Scale bar is 100 μm.

(**D**) Quantification of NTCP expression in HepaRG^C^ and HepaRG^EDEM3^ by In-cell ELISA. The results show the data and standard deviations from two independent experiments performed in triplicate biological samples. The data were analyzed using unpaired t*-*test; ns, non-statistical relevance.

(**E**) HBV DNA was isolated from virus particles internalized in HepaRG^EDEM3^ and HepaRG^C^ at 24 h post-infection and quantified by real-time PCR. The data and standard deviations from three independent experiments are shown. Statistical analysis was performed using unpaired t-test; ns, non-statistical relevance.


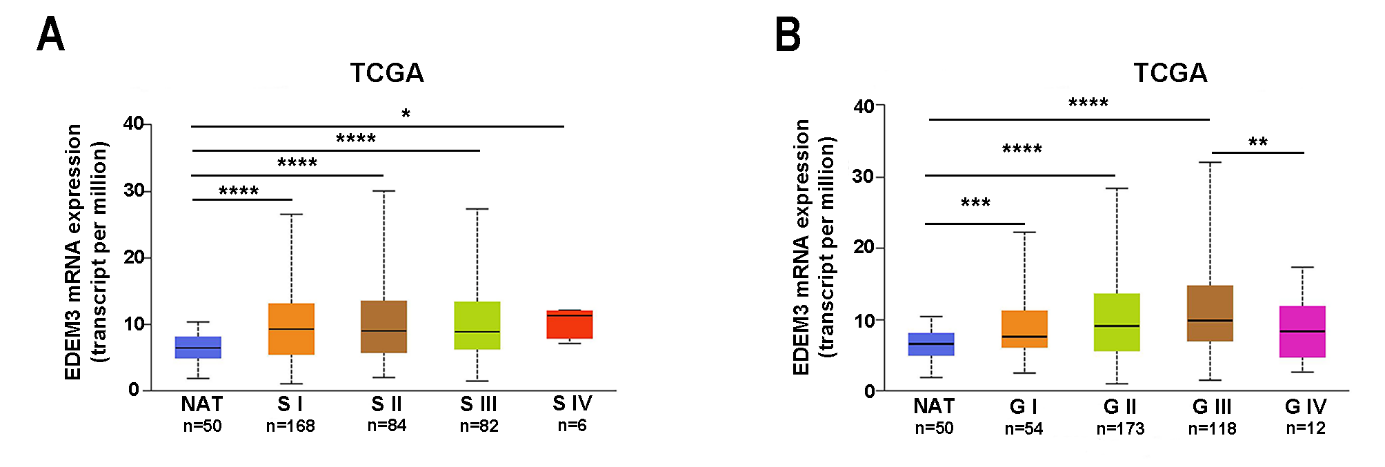


**Supplementary Figure 6.** EDEM3 expression correlates with pathological stage and tumor grade in HCC patients.

The correlation of EDEM3 gene expression with HCC clinical stage (**A**) and tumor grade (**B**), in the TCGA-LIHC cohort was analyzed using the UALCAN platform; S, stage, G, tumor grade. (*, *p* < 0.05, **, *p* < 0.01; ***, *p* < 0.001; ****, *p* < 0.0001.

Supplementary Table 1. Demographic and clinical-pathological information of HCC patients

| **Characteristic** | **N = 50**^1^ |  |
| --- | --- | --- |
| Age (years) |  |  |
| Median (IQR) | 64 (60, 68) |  |
| Gender |  |  |
| Female | 11 (22%) |  |
| Male | 39 (78%) |  |
| Edmondson-Steiner grading |  |  |
| ES I-II | 6 (12%) |  |
| ES II-III | 40 (80%) |  |
| ES III-IV | 4 (8.0%) |  |
| HBV infection (0=no, 1=yes) |  |  |
| 0 | 25 (50%) |  |
| 1 | 25 (50%) |  |
| Mellitus Diabetes (0=no, 1=yes) |  |  |
| 0 | 34 (68%) |  |
| 1 | 16 (32%) |  |
| Cirrhosis status (0=no, 1=yes) |  |  |
| 0 | 23 (46%) |  |
| 1 | 27 (54%) |  |
| AST (U/L) |  |  |
| Median (IQR) | 51 (37, 87) |  |
| ALT (U/L) |  |  |
| Median (IQR) | 57 (37, 89) |  |
| Albumin (g/dL) |  |  |
| Median (IQR) | 3.80 (3.05, 4.26) |  |
| AFP (ng/mL) |  |  |
| Median (IQR) | 13 (6, 60) |  |
| ^1^Median (IQR) or Frequency (%)  IQR, interquartile range; AST, aspartate aminotransferase; ALT, Alanine transaminase; AFP, Alpha Fetoprotein; | | |

Supplementary Table 2. Demographic and clinical-pathological information of patients with liver-related benign pathologies

| **Characteristic** | **N** | **N = 10**^1^ |
| --- | --- | --- |
| Age (years) | 10 |  |
| Mean (SD) |  | 43 (19) |
| Median (IQR) |  | 37 (30, 53) |
| Gender | 10 |  |
| Female |  | 9 (90%) |
| Male |  | 1 (10%) |
| HBV status | 10 |  |
| 0 |  | 10 (100%) |
| Mellitus Diabetes | 10 |  |
| 0 |  | 9 (90%) |
| 1 |  | 1 (10%) |
| Cirrhosis status | 10 |  |
| 0 |  | 10 (100%) |
| AST (U/L) | 10 |  |
| Mean (SD) |  | 22.8 (7.0) |
| Median (IQR) |  | 21.5 (18.8, 22.0) |
| ALT (U/L) | 9 |  |
| Mean (SD) |  | 24 (14) |
| Median (IQR) |  | 21 (16, 25) |
| Albumin (g/dL) | 8 |  |
| Mean (SD) |  | 4.56 (0.42) |
| Median (IQR) |  | 4.50 (4.20, 4.80) |
| AFP (ng/mL) | 6 |  |
| Mean (SD) |  | 3.05 (2.45) |
| Median (IQR) |  | 2.75 (0.95, 4.48) |
| ^1^Median (IQR) or Frequency (%)  IQR, interquartile range; AST, aspartate aminotransferase; ALT, Alanine transaminase; AFP, Alpha Fetoprotein; | | |

Supplementary Table 3. Sequences of primers used for RT real-time PCR

| **Primer** | **Sequences (5'--3')** |
| --- | --- |
| EDEM3 | F: GGCTTGGTGGCTTCGGGAAA |
| EDEM3 | R: ACATTGCTGGACGCTGGTGG |
| ATF6 | F: CTTTTAGCCCGGGACTCTTT |
| ATF6 | R: TCAGCAAAGAGAGCAGAATCC |
| LC3 | F: GCCTTCTTCCTGCTGGTGAA |
| LC3 | R: TCCTGCTCGTAGATGTCCGC |
| ATG3 | F: CGGAAGCCGTTAAAGAGATCA |
| ATG3 | R: CTGCAGCTTCTCCTTCATCTT |
| TBP | F: CCCATGACTCCCATGACC |
| TBP | R: TTTACAACCAAGATTCACTGTGG |
| GAPDH | F: ACCCACTCCTCCACCTTTGA |
| GAPDH | R: CTGTTGCTGTAGCCAAATTCGT |
| β-actin | F: GAAAATCTGGCACCACACCTTC |
| β-actin | R: CTCGGTGAGGATCTTCATGAGG |
| aldolase B | F: GAAGAAAGGAGCTCTCAGAAATTG |
| aldolase B | R: AGAAGAGGATTTCTCGGAACTG |
| albumin | F: TTTCTCTTTAGCTCGGCTTATTC |
| albumin | R: ATGATCTTCAAATGGACACTGC |
| HBV | F: TCCAGGATCCTCAACAACCAGCACG |
| HBV | R: TGGCCCCCAATACCACATCATCC |

F, forward primer; R, reverse primer.
